# Scaling up strategies of the chronic respiratory disease programme of the European Innovation Partnership on Active and Healthy Ageing (Action Plan B3: Area 5)

**DOI:** 10.1186/s13601-016-0116-9

**Published:** 2016-07-29

**Authors:** J. Bousquet, J. Farrell, G. Crooks, P. Hellings, E. H. Bel, M. Bewick, N. H. Chavannes, J. Correia de Sousa, A. A. Cruz, T. Haahtela, G. Joos, N. Khaltaev, J. Malva, A. Muraro, M. Nogues, S. Palkonen, S. Pedersen, C. Robalo-Cordeiro, B. Samolinski, T. Strandberg, A. Valiulis, A. Yorgancioglu, T. Zuberbier, A. Bedbrook, W. Aberer, M. Adachi, A. Agusti, C. A. Akdis, M. Akdis, J. Ankri, A. Alonso, I. Annesi-Maesano, I. J. Ansotegui, J. M. Anto, S. Arnavielhe, H. Arshad, C. Bai, I. Baiardini, C. Bachert, A. K. Baigenzhin, C. Barbara, E. D. Bateman, B. Beghé, A. Ben Kheder, K. S. Bennoor, M. Benson, K. C. Bergmann, T. Bieber, C. Bindslev-Jensen, L. Bjermer, H. Blain, F. Blasi, A. L. Boner, M. Bonini, S. Bonini, S. Bosnic-Anticevitch, L. P. Boulet, R. Bourret, P. J. Bousquet, F. Braido, A. H. Briggs, C. E. Brightling, J. Brozek, R. Buhl, P. G. Burney, A. Bush, F. Caballero-Fonseca, D. Caimmi, M. A. Calderon, P. M. Calverley, P. A. M. Camargos, G. W. Canonica, T. Camuzat, K. H. Carlsen, W. Carr, A. Carriazo, T. Casale, A. M. Cepeda Sarabia, L. Chatzi, Y. Z. Chen, R. Chiron, E. Chkhartishvili, A. G. Chuchalin, K. F. Chung, G. Ciprandi, I. Cirule, L. Cox, D. J. Costa, A. Custovic, R. Dahl, S. E. Dahlen, U. Darsow, G. De Carlo, F. De Blay, T. Dedeu, D. Deleanu, E. De Manuel Keenoy, P. Demoly, J. A. Denburg, P. Devillier, A. Didier, A. T. Dinh-Xuan, R. Djukanovic, D. Dokic, H. Douagui, G. Dray, R. Dubakiene, S. R. Durham, M. S. Dykewicz, Y. El-Gamal, R. Emuzyte, L. M. Fabbri, M. Fletcher, A. Fiocchi, A. Fink Wagner, J. Fonseca, W. J. Fokkens, F. Forastiere, P. Frith, M. Gaga, A. Gamkrelidze, J. Garces, J. Garcia-Aymerich, B. Gemicioğlu, J. E. Gereda, S. González Diaz, M. Gotua, I. Grisle, L. Grouse, Z. Gutter, M. A. Guzmán, L. G. Heaney, B. Hellquist-Dahl, D. Henderson, A. Hendry, J. Heinrich, D. Heve, F. Horak, J. O’. B. Hourihane, P. Howarth, M. Humbert, M. E. Hyland, M. Illario, J. C. Ivancevich, J. R. Jardim, E. J. Jares, C. Jeandel, C. Jenkins, S. L. Johnston, O. Jonquet, K. Julge, K. S. Jung, J. Just, I. Kaidashev, M. R. Kaitov, O. Kalayci, A. F. Kalyoncu, T. Keil, P. K. Keith, L. Klimek, B. Koffi N’Goran, V. Kolek, G. H. Koppelman, M. L. Kowalski, I. Kull, P. Kuna, V. Kvedariene, B. Lambrecht, S. Lau, D. Larenas-Linnemann, D. Laune, L. T. T. Le, P. Lieberman, B. Lipworth, J. Li, K. Lodrup Carlsen, R. Louis, W. MacNee, Y. Magard, A. Magnan, B. Mahboub, A. Mair, I. Majer, M. J. Makela, P. Manning, S. Mara, G. D. Marshall, M. R. Masjedi, P. Matignon, M. Maurer, S. Mavale-Manuel, E. Melén, E. Melo-Gomes, E. O. Meltzer, A. Menzies-Gow, H. Merk, J. P. Michel, N. Miculinic, F. Mihaltan, B. Milenkovic, G. M. Y. Mohammad, M. Molimard, I. Momas, A. Montilla-Santana, M. Morais-Almeida, M. Morgan, R. Mösges, J. Mullol, S. Nafti, L. Namazova-Baranova, R. Naclerio, A. Neou, H. Neffen, K. Nekam, B. Niggemann, G. Ninot, T. D. Nyembue, R. E. O’Hehir, K. Ohta, Y. Okamoto, K. Okubo, S. Ouedraogo, P. Paggiaro, I. Pali-Schöll, P. Panzner, N. Papadopoulos, A. Papi, H. S. Park, G. Passalacqua, I. Pavord, R. Pawankar, R. Pengelly, O. Pfaar, R. Picard, B. Pigearias, I. Pin, D. Plavec, D. Poethig, W. Pohl, T. A. Popov, F. Portejoie, P. Potter, D. Postma, D. Price, K. F. Rabe, F. Raciborski, F. Radier Pontal, S. Repka-Ramirez, S. Reitamo, S. Rennard, F. Rodenas, J. Roberts, J. Roca, L. Rodriguez Mañas, C. Rolland, M. Roman Rodriguez, A. Romano, J. Rosado-Pinto, N. Rosario, L. Rosenwasser, M. Rottem, D. Ryan, M. Sanchez-Borges, G. K. Scadding, H. J. Schunemann, E. Serrano, P. Schmid-Grendelmeier, H. Schulz, A. Sheikh, M. Shields, N. Siafakas, Y. Sibille, T. Similowski, F. E. R. Simons, J. C. Sisul, I. Skrindo, H. A. Smit, D. Solé, T. Sooronbaev, O. Spranger, R. Stelmach, P. J. Sterk, J. Sunyer, C. Thijs, T. To, A. Todo-Bom, M. Triggiani, R. Valenta, A. L. Valero, E. Valia, E. Valovirta, E. Van Ganse, M. van Hage, O. Vandenplas, T. Vasankari, B. Vellas, J. Vestbo, G. Vezzani, P. Vichyanond, G. Viegi, C. Vogelmeier, T. Vontetsianos, M. Wagenmann, B. Wallaert, S. Walker, D. Y. Wang, U. Wahn, M. Wickman, D. M. Williams, S. Williams, J. Wright, B. P. Yawn, P. K. Yiallouros, O. M. Yusuf, A. Zaidi, H. J. Zar, M. E. Zernotti, L. Zhang, N. Zhong, M. Zidarn, J. Mercier

**Affiliations:** 1 CHRU, University Hospital, 371 Avenue du Doyen Gaston Giraud, 34295 Montpellier Cedex 5, France; 2 MACVIA-LR, Contre les MAladies Chroniques pour un VIeillissement Actif en Languedoc Roussilon, European Innovation Partnership on Active and Healthy Ageing Reference Site, Montpellier, France; 3 INSERM, VIMA: Ageing and Chronic Diseases, Epidemiological and Public Health Approaches, U1168, Paris, France; 4 UVSQ, UMR-S 1168, Université Versailles St-Quentin-en-Yvelines, Paris, France; 5 Department of Health, Social Services and Public Safety, Belfast, Northern Ireland, UK; 6 EIP on AHA, European Innovation Partnership on Active and Healthy Ageing, Reference Site, Scottish Centre for Telehealth and Telecare, NHS 24, Glasgow, UK; 7 Laboratory of Clinical Immunology, Department of Microbiology and Immunology, KU Leuven, Louvain, Belgium; 8 European Academy of Allergy and Clinical Immunology, Zurich, Switzerland; 9 Department of Respiratory Medicine, Academic Medical Center (AMC), University of Amsterdam, Amsterdam, The Netherlands; 10 European Respiratory Society, Lausanne, Switzerland; 11 iQ4U Consultants Ltd, London, UK; 12 Department of Public Health and Primary Care, Leiden University Medical Center, Leiden, The Netherlands; 13 Global Alliance Against Chronic Respiratory Diseases (GARD), Cape Town, South Africa; 14 International Primary Care Respiratory Group, Westhill, UK; 15 Life and Health Sciences Research Institute, ICVS, School of Health Sciences, University of Minho, Braga, Portugal; 16 ProAR – Nucleo de Excelencia em Asma, Federal University of Bahia, Bahia, Brazil; 17 GARD Executive Committee, Bahia, Brazil; 18 EIP on AHA Commitment for Action, Lisbon, Portugal; 19 Skin and Allergy Hospital, Helsinki University Hospital, Helsinki, Finland; 20 Department of Respiratory Medicine, Ghent University Hospital, Ghent, Belgium; 21 Faculty of Medicine, University of Coimbra, Coimbra, Portugal; 22 Ageing@Coimbra Reference Site, Coimbra, Portugal; 23 Food Allergy Referral Centre Veneto Region, Department of Women and Child Health, Padua General University Hospital, Padua, Italy; 24 Caisse Assurance Retraite et Santé Au Travail Languedoc-Roussillon (CARSAT-LR), 34000 Montpellier, France; 25 EFA European Federation of Allergy and Airways Diseases Patients’ Associations, Brussels, Belgium; 26 University of Southern Denmark, Kolding, Denmark; 27 Centre of Pneumology, Coimbra University Hospital, Coimbra, Portugal; 28 Department of Prevention of Environmental Hazards and Allergology, Medical University of Warsaw, Warsaw, Poland; 29 Helsinki University, Helsinki University Hospital, Helsinki, Finland; 30 Center for Life Course Health Research, University of Oulu, Oulu, Finland; 31 European Union GeriatricMedicine Society, EUGMS, Oslo, Norway; 32 Center of Quality of Life Research, Vilnius University Clinic of Children’s Diseases, Vilnius University Public Health Institute, Vilnius, Lithuania; 33 European Association of Pediatrics (EAP/UEMS-SP), Brussels, Belgium; 34 Department of Pulmonology, Celal Bayar University, Manisa, Turkey; 35 Turkish Thoracic Society, Antalya, Turkey; 36 Allergy-Centre-Charité at the Department of Dermatology, Charité - Universitätsmedizin Berlin, Berlin, Germany; 37 Secretary General of the Global Allergy and Asthma European Network (GA²LEN), Berlin, Germany; 38 Department of Dermatology, Medical University of Graz, Graz, Austria; 39 Department of Clinical Research Center, International University of Health and Welfare/Sanno Hospital, Tokyo, Japan; 40 Thorax Institute, Hospital Clinic, IDIBAPS, University of Barcelona, Barcelona, Spain; 41 CIBER Enfermedades Respiratorias, Barcelona, Spain; 42 Swiss Institute of Allergy and Asthma Research (SIAF), University of Zurich, Davos, Switzerland; 43 EPAR U707 INSERM, Paris, France; 44 Department of Allergy and Immunology, Hospital Quirón Bizkaia, Erandio, Spain; 45 Centre for Research in Environmental Epidemiology (CREAL), Barcelona, Spain; 46 Hospital del Mar Research Institute (IMIM), Barcelona, Spain; 47 CIBER Epidemiología y Salud Pública (CIBERESP), Barcelona, Spain; 48 Department of Experimental and Health Sciences, University of Pompeu Fabra (UPF), Barcelona, Spain; 49 Digi Health, Montpellier, France; 50 David Hide Asthma and Allergy Research Centre, Isle of Wight, UK; 51 Shanghai Respiratory Research Institute, Vice President of Respiratory Society, Chinese Medical Association, China and Chinese Alliance Against Lung Cancer, Shanghai, China; 52 Allergy and Respiratory Diseases Clinic, DIMI, IRCCS AOU San Martino-IST, University of Genoa, Genoa, Italy; 53 Upper Airways Research Laboratory, ENT Department, Ghent University Hospital, Ghent, Belgium; 54 EuroAsian Respiratory Society, Astana City, Kazakhstan; 55 Faculdade de Medicina de Lisboa, Portuguese National Programme for Respiratory Diseases (PNDR), Lisbon, Portugal; 56 Department of Medicine, University of Cape Town, Cape Town, South Africa; 57 Section of Respiratory Disease, Department of Oncology, Haematology and Respiratory Diseases, University of Modena and Reggio Emilia, Modena, Italy; 58 Service de pneumologie IV, hôpital Abderrahman Mami, Ariana, 2080 Tunis, Tunisia; 59 Department of Respiratory Medicine, National Institute of Diseases of the Chest and Hospital, Dhaka, Bangladesh; 60 Centre for Individualized Medicine, Department of Pediatrics, Faculty of Medicine, LInköping University, Linköping, Sweden; 61 Department of Dermatology and Allergy, Rheinische Friedrich-Wilhelms-University Bonn, Bonn, Germany; 62 Department of Dermatology and Allergy Centre, Odense University Hospital, Odense, Denmark; 63 Department of Respiratory Medicine and Allergology, University Hospital, Lund, Sweden; 64 Department of Geriatrics, Montpellier University Hospital, Montpellier, France; 65 EA 2991, Euromov, University Montpellier, Montpellier, France; 66 Department of Pathophysiology and Transplantation, IRCCS Fondazione Ca’Granda Ospedale Maggiore Policlinico, University of Milan, Via F. Sforza 35, Milan, Italy; 67 Pediatric Department, University of Verona Hospital, Verona, Italy; 68 Department of Public Health and Infectious Diseases, Sapienza University of Rome, Rome, Italy; 69 Second University of Naples and Institute of Translational Medicine, Italian National Research Council, Naples, Italy; 70 Woolcock Institute of Medical Research, University of Sydney and Sydney Local Health District, Glebe, NSW Australia; 71 Quebec Heart and Lung Institute, Laval University, Québec City, QC Canada; 72 Directeur Général Adjoint, Montpellier University Hospital, Montpellier, France; 73 Health Economics and Health Technology Assessment, Institute of Health and Wellbeing, University of Glasgow, Glasgow, UK; 74 Respiratory Biomedical Unit, Institute of Lung Health, University Hospitals of Leicester NHS Trust, Leicestershire, UK; 75 Department of Infection, Immunity and Inflammation, University of Leicester, Leicester, UK; 76 Department of Clinical Epidemiology and Biostatistics, McMaster University, HSC Room 2C16, 1280 Main Street West Hamilton, Hamilton, Canada; 77 Universitätsmedizin der Johannes Gutenberg-Universität Mainz, Mainz, Germany; 78 National Heart and Lung Institute, Imperial College, London, London, UK; 79 Wellcome Centre for Global Health, Imperial College, London, London, UK; 80 MRC-PHE Centre for Environment and Health, Imperial College, London, London, UK; 81 Imperial College and Royal Brompton Hospital, London, UK; 82 Centro Medico Docente La Trinidad, Caracas, Venezuela; 83 Department of Respiratory Diseases, Montpellier University Hospital, Montpellier, France; 84 National Heart and Lung Institute, Imperial College London, Royal Brompton Hospital NHS, London, UK; 85 Institute of Ageing and Chronic Disease, University of Liverpool and University Hospital Aintree, Liverpool, UK; 86 Department of Pediatrics, Medical School, Federal University of Minas Gerais, Belo Horizonte, Brazil; 87 Région Languedoc Roussillon, Montpellier, France; 88 Department of Paediatrics, Oslo University Hospital and University of Oslo, Oslo, Norway; 89 Allergy and Asthma Associates of Southern California, Mission Viejo, CA USA; 90 Regional Ministry of Equality, Health and Social Policies of Andalusia, Seville, Spain; 91 Division of Allergy/Immunology, University of South Florida, Tampa, FL USA; 92 Allergy and Immunology Laboratory, Metropolitan University, Simon Bolivar University, Barranquilla, Colombia; 93 Asma e Immunologia, SLaai, Sociedad Latinoamericana de Allergia, Barranquilla, Colombia; 94 Department of Social Medicine, Faculty of Medicine, University of Crete, PO Box 2208, Heraklion, 71003 Crete Greece; 95 National Cooperative Group of Paediatric Research on Asthma, Asthma Clinic and Education Center of the Capital Institute of Pediatrics, Peking and Center for Asthma Research and Education, Beijing, China; 96 Chachava Clinic, David Tvildiani Medical University-AIETI Medical School, Grigol Robakidze University, Tbilisi, Georgia; 97 Pulmonolory Research Institute FMBA, Moscow, Russia; 98 National Heart and Lung Institute, Imperial College, London, London, UK; 99 Medicine Department, IRCCS-Azienda Ospedaliera Universitaria San Martino, Genoa, Italy; 100 Latvian Allergy Association, Riga, Latvia; 101 Department of Medicine, Nova Southeastern University, Davie, FL USA; 102 Department of Paediatrics, Imperial College London, London, UK; 103 The Centre for Allergy Research, The Institute of Environmental Medicine, Karolinska Institutet, Stockholm, Sweden; 104 Department of Dermatology and Allergy, Technische Universität München, Munich, Germany; 105 ZAUM-Center for Allergy and Environment, Helmholtz Center Munich, Technische Universität München, Munich, Germany; 106 Allergy Division, Chest Disease Department, University Hospital of Strasbourg, Strasbourg, France; 107 EUREGHA, European Regional and Local Health Association, Brussels, Belgium; 108 University of Edinburgh, Edinburgh, UK; 109 Allergology and Immunology Discipline, “Iuliu Hatieganu” University of Medicine and Pharmacy, Cluj-Napoca, Romania; 110 Kronikgune, Bilbao, Basque Region Spain; 111 Department of Medicine, Division of Clinical Immunology and Allergy, McMaster University, Hamilton, ON Canada; 112 Laboratoire de Pharmacologie Respiratoire UPRES EA220, Hôpital Foch, Suresnes Université Versailles Saint-Quentin, Suresnes, France; 113 Respiratory Diseases Department, Rangueil-Larrey Hospital, Toulouse, France; 114 Service de physiologie respiratoire, Hôpital Cochin, Université Paris-Descartes, Assistance publique-Hôpitaux de Paris, Paris, France; 115 NIHR Southampton Respiratory Biomedical Research Unit, Faculty of Medicine, University Southampton, Southampton, UK; 116 Medical Faculty Skopje, University Clinic of Pulmology and Allergy, Skopje, Republic Macedonia; 117 Service de Pneumo-Allergologie, Centre Hospitalo-Universitaire de Béni-Messous, Algers, Algeria; 118 Ecole des Mines, Alès, France; 119 Medical Faculty, Vilnius University, Vilnius, Lithuania; 120 Allergy and Clinical Immunology Section, National Heart and Lung Institute, Imperial College London, London, UK; 121 Section of Allergy and Immunology, Saint Louis University School of Medicine, Saint Louis, MO USA; 122 Pediatric Allergy and Immunology Unit, Ain Shams University, Cairo, Egypt; 123 Clinic of Children’s Diseases, Faculty of Medicine, Vilnius University, Vilnius, Lithuania; 124 Modena University, Modena, Italy; 125 Education for Health, Warwick, UK; 126 Division of Allergy, Department of Pediatric Medicine, The Bambino Gesù Children’s Research Hospital Holy See, Rome, Italy; 127 Global Allergy and Asthma Platform (GAAPP), Altgasse 8-10, 1130 Vienna, Austria; 128 Center for Health Technology and Services Research - CINTESIS, Faculdade de Medicina, Universidade do Porto, Porto, Portugal; 129 Allergy Unit, CUF Porto Instituto & Hospital, Porto, Portugal; 130 Department of Otorhinolaryngology, Academic Medical Centre, Amsterdam, The Netherlands; 131 Department of Epidemiology, Regional Health Service Lazio Region, Rome, Italy; 132 Repatriation General Hospital, Adelaide, SOUTH AUSTRALIA Australia; 133 Athens Chest Hospital, Athens, Greece; 134 National Center for Disease Control and Public Health of Georgia, Tbilisi, Georgia; 135 Polibienestar Research Institute, University of Valencia, Valencia, Spain; 136 Department of Pulmonary Diseases, Cerrahpasa Faculty of Medicine, Istanbul University, Istanbul, Turkey; 137 Allergy and Immunology Division, Clinica Ricardo Palma, Lima, Peru; 138 Universidad Autónoma de Nuevo León, San Nicolás De La Garza, Mexico; 139 Center of Allergy and Immunology, Georgian Association of Allergology and Clinical Immunology, Tbilisi, Georgia; 140 Latvian Association of Allergists, Center of Tuberculosis and Lung Diseases, Riga, Latvia; 141 Faculty of the Department of Neurology, University of Washington School of Medicine, St. Louis, MO USA; 142 University Hospital Olomouc – National eHealth Centre, Olomouc, Czech Republic; 143 Immunology and Allergy Division, Clinical Hospital, University of Chile, Santiago, Chile; 144 Centre for Infection and Immunity, School of Medicine, Dentistry and Biomedical Sciences, Queen’s University Belfast, Belfast, UK; 145 Department of Respiratory Diseases, Odense University Hospital, Odense, Denmark; 146 NHS Scotland, Edinburgh, Scotland, UK; 147 Institute of Epidemiology I, German Research Centre for Environmental Health, Helmholtz Zentrum München, Neuherberg, Germany; 148 Agence Régionale de Santé, 34067 Montpellier Cedex 2, France; 149 Vienna Challenge Chamber, Vienna, Austria; 150 Department of Paediatrics and Child Health, University College Cork, Cork, Ireland; 151 University of Southampton Faculty of Medicine, University Hospital Southampton, Southampton, UK; 152 Service de Pneumologie, Hôpital Bicêtre, Inserm UMR_S999, Université Paris-Sud, Le Kremlin Bicêtre, France; 153 School of Psychology, Plymouth University, Plymouth, UK; 154 Federico II University Hospital/Campania RS, Naples, Italy; 155 Servicio de Alergia e Immunologia, Clinica Santa Isabel, Buenos Aires, Argentina; 156 Universidade Federal de Sao Paulo, São Paulo, Brazil; 157 Libra Foundation, Buenos Aires, Argentina; 158 The George Institute for Global Health, The University of Sydney, Camperdown, Australia; 159 Airway Disease Infection Section, National Heart and Lung Institute, Imperial College, London, London, UK; 160 MRC and Asthma UK Centre in Allergic Mechanisms of Asthma, London, UK; 161 Medical Commission, Montpellier University Hospital, Montpellier, France; 162 Children’s Clinic of Tartu University Hospital, Tartu, Estonia; 163 Hallym University College of Medicine, Hallym University Sacred Heart Hospital, Gyeonggi-Do, South Korea; 164 Allergology Department, Centre de l’Asthme et des Allergies, Hôpital d’Enfants Armand-Trousseau (APHP), Paris, France; 165 Sorbonne Universités, UPMC Univ Paris 06, UMR_S 1136, Institut Pierre Louis d’Epidémiologie et de Santé Publique, Equipe EPAR, 75013 Paris, France; 166 Ukrainian Medical Stomatological Academy, Poltava, Ukraine; 167 Federal Medicobiological Agency, Laboratory of Molecular Immunology, Institute of Immunology, National Research Center, Moscow, Russian Federation; 168 Pediatric Allergy and Asthma Unit, Hacettepe University School of Medicine, Ankara, Turkey; 169 Immunology and Allergy Division, Department of Chest Diseases, School of Medicine, Hacettepe University, Ankara, Turkey; 170 Institute of Social Medicine, Epidemiology and Health Economics, Charité - Universitätsmedizin Berlin, Berlin, Germany; 171 Institute for Clinical Epidemiology and Biometry, University of Würzburg, Würzburg, Germany; 172 Department of Medicine, McMaster University, Health Sciences Centre 3V47, 1280 Main Street West, Hamilton, Canada; 173 Center for Rhinology and Allergology, Wiesbaden, Germany; 174 Société de Pneumologie de Langue Française, Espace francophone de Pneumologie, Paris, France; 175 Department of Respiratory Medicine, Faculty of Medicine and Dentistry, University Hospital Olomouc, Olomouc, Czech Republic; 176 GRIACResearch Institute, Department of Pediatric Pulmonology and Pediatric Allergology, Beatrix Children’s Hospital, University Medical Center Groningen, University of Groningen, Groningen, The Netherlands; 177 Department of Immunology, Rheumatology and Allergy, Medical University of Lodz, and HARC, Lodz, Poland; 178 Sachs’ Children’s Hospital, Stockholm, Sweden; 179 Institute of Environmental Medicine, Karolinska Institutet, Stockholm, Sweden; 180 Division of Internal Medicine, Asthma and Allergy, Barlicki University Hospital, Medical University of Lodz, Lodz, Poland; 181 Clinic of Infectious, Chest Diseases, Dermatology and Allergology, Vilnius University, Vilnius, Lithuania; 182 VIB Inflammation Research Center, Ghent University, Ghent, Belgium; 183 Department for Pediatric Pneumology and Immunology, Charité Medical University, Berlin, Germany; 184 Clínica de Alergia, Asma y Pediatría, Hospital Médica Sur, Ciudad De México, Mexico; 185 University of Medicine and Pharmacy, Hochiminh City, Vietnam; 186 Divisions of Allergy and Immunology, Departments of Internal Medicine and Pediatrics, University of Tennessee College of Medicine, Germantown, TN USA; 187 Scottish Centre for Respiratory Research, Cardiovascular and Diabetes Medicine, Medical Research Institute, Ninewells Hospital, University of Dundee, Dundee, UK; 188 State Key Laboratory of Respiratory Diseases, Guangzhou Institute of Respiratory Disease, The First Affiliated Hospital of Guangzhou Medical University, Guangzhou, 510120 China; 189 Department of Paediatrics, Oslo University Hospital, Oslo, Norway; 190 Faculty of Medicine, Institute of Clinical Medicine, University of Oslo, Oslo, Norway; 191 Department of Pulmonary Medicine, CHU Sart-Tilman, Liege, Belgium; 192 Queen’s Medical Research Institute, University of Edinburgh, Edinburgh, UK; 193 Service de Pneumo-allergologie, Hôpital Saint-Joseph, Paris, France; 194 Service de Pneumologie, UMR INSERM, UMR1087and CNR 6291, l’institut du thorax, University of Nantes, Nantes, France; 195 Department of Pulmonary Medicine, Rashid Hospital, Dubai, UAE; 196 Scottish Government Health Department, eHealth and Pharmaceuticals, Edinburgh, UK; 197 Department of Respiratory Medicine, University of Bratislava, Bratislava, Slovakia; 198 Department of Medicine (RCSI), Bon Secours Hospital, Glasnevin, Dublin, Ireland; 199 Cardiovascular and Thoracic Department, AOU Città della Salute e della Scienza di Torino, Turin, Italy; 200 Division of Clinical Immunology and Allergy, Laboratory of Behavioral Immunology Research, The University of Mississippi Medical Center, Jackson, MS USA; 201 Respiratory Medicine Research, Shahid Beheshti University of Medical Sciences, Tehran, Iran; 202 VingCard Elsafe, Moss, Norway; 203 Allergie-Centrum-Charité at the Department of Dermatology and Allergy, Charité - Universitätsmedizin Berlin, Berlin, Germany; 204 Department of Paediatrics, Maputo Central Hospital, Maputo, Mozambique; 205 Institute of Environmental Medicine, Karolinska Institutet, Stockholm, Sweden; 206 PNDR/Portuguese National Programme for Respiratory Diseases, Lisbon, Portugal; 207 Allergy and Asthma Medical Group and Research Center, San Diego, CA USA; 208 Royal Brompton Hospital, London, UK; 209 Hautklinik - Klinik für Dermatologie & Allergologie, Universitätsklinikum der RWTH Aachen, Aachen, Germany; 210 Croatian Pulmonary Society, Zagreb, Croatia; 211 National Institute of Pneumology M. Nasta, Bucharest, Romania; 212 Faculty of Medicine, University of Belgrade, Belgrade, Serbia; 213 Serbian Association for Asthma and COPD, Belgrade, Serbia; 214 National Center for Research in Chronic Respiratory Diseases, Tishreen University School of Medicine, Latakia, Syria; 215 Département de Pharmacologie, CHU de Bordeaux, Universite Bordeaux, INSERM U657, Bordeaux Cedex, France; 216 Department of Public Health and Biostatistics, EA 4064, Paris Descartes University, Paris, France; 217 Paris Municipal Department of Social Action, Childhood, and Health, Paris, France; 218 Aura Andalucia, Andalucía, Spain; 219 Allergy and Clinical Immunology Department, Hospital CUF-Descobertas, Lisbon, Portugal; 220 National Clinical Director for Respiratory Services, NHS England, Leeds, England, UK; 221 Institute of Medical Statistics, Informatics and Epidemiology, Medical Faculty, University of Cologne, Cologne, Germany; 222 Unitat de Rinologia i Clínica de l’Olfacte, Servei d’ORL, Hospital Clínic, Clinical and Experimental Respiratory Immunoallergy, IDIBAPS, Barcelona, Spain; 223 Mustapha Hospital, Algers, Algeria; 224 Scientific Centre of Children’s Health Under the Russian Academy of Medical Sciences, Moscow, Russia; 225 Section of Otolaryngology-Head and Neck Surgery, The University of Chicago Medical Center and The Pritzker School of Medicine, The University of Chicago, Chicago, IL USA; 226 Hospital de Niños Orlando Alassia, Santa Fe, Argentina; 227 Hospital of the Hospitaller Brothers in Buda, Budapest, Hungary; 228 Pediatric Pneumology and Immunology, Charité Universitätsmedizin Berlin, Berlin, Germany; 229 EA4556 Epsylon, Université Montpellier 1, Montpellier, France; 230 ENT Department, University Hospital of Kinshasa, Kinshasa, Congo; 231 Department of Allergy, Immunology and Respiratory Medicine, Alfred Hospital and Central Clinical School, Monash University, Melbourne, VIC Australia; 232 Department of Immunology, Monash University, Melbourne, VIC Australia; 233 National Hospital Organization, Tokyo National Hospital, Tokyo, Japan; 234 Department of Otorhinolaryngology, Chiba University Hospital, Chiba, Japan; 235 Department of Otolaryngology, Nippon Medical School, Tokyo, Japan; 236 Centre Hospitalier Universitaire Pédiatrique Charles de Gaulle, Ouagadougou, Burkina Faso; 237 Cardio-Thoracic and Vascular Department, University Hospital of Pisa, Pisa, Italy; 238 Department of Comparative Medicine, Messerli, Research Institute of the University of Veterinary Medicine and Medical University, Vienna, Austria; 239 Department of Immunology and Allergology, Faculty of Medicine and Faculty Hospital in Pilsen, Charles University in Prague, Pilsen, Czech Republic; 240 Center for Pediatrics and Child Health, Institute of Human Development, Royal Manchester Children’s Hospital, University of Manchester, Manchester, M13 9WL UK; 241 Allergy Department, 2nd Pediatric Clinic, Athens General Children’s Hospital “P&A Kyriakou”, University of Athens, Athens, 11527 Greece; 242 Respiratory Medicine, Department of Medical Sciences, University of Ferrara, Ferrara, Italy; 243 Department of Allergy and Clinical Immunology, Ajou University School of Medicine, Suwon, South Korea; 244 Nuffield Department of Medicine, University of Oxford, Oxford, UK; 245 Department of Pediatrics, Nippon Medical School, Tokyo, Japan; 246 Department of Health, Social Services and Public Safety, Belfast, Northern Ireland, UK; 247 Center for Rhinology and Allergology, Wiesbaden, Germany; 248 Department of Otorhinolaryngology, Head and Neck Surgery, Universitätsmedizin Mannheim, Medical Faculty Mannheim, Heidelberg University, Mannheim, Germany; 249 Conseil Général de l’Economie. Ministère de l’Economie, de l’Industrie et du Numérique, Paris, France; 250 Département de pédiatrie, CHU de Grenoble, BP 217, 38043 Grenoble Cedex 9, France; 251 Children’s Hospital Srebrnjak, Zagreb, School of Medicine, University J.J. Strossmayer, Osijek, Croatia; 252 Im GerontoLab Europe - Europäische Vereinigung für Vitalität und Aktives Altern (eVAA) e.V., Leipzig, Germany; 253 Karl Landsteiner Institute for Clinical and Experimental Pneumology, Hietzing Hospital, Wolkersbergenstraße 1, 1130 Vienna, Austria; 254 Clinic of Allergy and Asthma, Medical University Sofia, 1Sv. Georgi Sofiyski St., 1431 Sofia, Bulgaria; 255 Allergy Diagnostic and Clinical Research Unit, University of Cape Town Lung Institute, Cape Town, South Africa; 256 Department of Pulmonary Medicine and Tuberculosis, GRIAC Research Institute, University Medical Center Groningen, University of Groningen, Groningen, The Netherlands; 257 Academic Centre of Primary Care, University of Aberdeen, Aberdeen, UK; 258 Research in Real-Life, Cambridge, UK; 259 LungenClinic Grosshansdorf, Airway Research Center North, Member of the German Center for Lung Research (DZL), Grosshansdorf, Germany; 260 Department of Medicine, Christian Albrechts University, Airway Research Center North, Member of the German Center for Lung Research (DZL), Kiel, Germany; 261 Conseil Départemental de l’Ordre des Pharmaciens, Maison des Professions Libérales, 34000 Montpellier, France; 262 SLAAI, Barranquilla, Colombia; 263 Division of Pulmonary, Critical Care, Sleep and Allergy, University of Nebraska Medical Center, Omaha, NE USA; 264 Salford, Royal NHS Foundation Trust and NHS England North, London, UK; 265 Hospital Universitario de Getafe-Servicio Madrileño de Salud, Madrid, Spain; 266 Association Asthme et Allergie, Paris, France; 267 Primary Care Respiratory Research Unit, Institutode Investigación Sanitaria de Palma IdisPa, Palma De Mallorca, Spain; 268 Allergy Unit, Complesso integrato Columbus, Rome, Italy; 269 Serviço de Imunoalergologia, Hospital da Luz, Lisbon, Portugal; 270 Hospital de Clinicas, University of Parana, Curitiba, PR Brazil; 271 Department of Allergy, Asthma, and Immunology, Children’s Mercy Hospitals and Clinics and Pediatrics, Medicine University of Misouri-Kansas City School of Medicine, Kansas City, MO USA; 272 Division of Allergy Asthma and Clinical Immunology, Emek Medical Center, Afula, Israel; 273 Woodbrook Medical Centre, Loughborough, UK; 274 Honorary Clinical Research Fellow, Allergy and Respiratory Research Group, The University of Edinburgh, Edinburgh, UK; 275 Allergy and Clinical Immunology Department, Centro Médico-Docente la, Trinidad and Clínica El Avila, 6a transversal Urb, Altamira, piso 8, consultorio 803, Caracas, 1060 Venezuela; 276 The Royal National TNE Hospital, University College London, London, UK; 277 Otolaryngology and Head and Neck Surgery, CHU Rangueil-Larrey, Toulouse, France; 278 Allergy Unit, Department of Dermatology, University Hospital of Zurich, Zurich, Switzerland; 279 Helmholtz Zentrum München/Institute of Epidemiology I, Neuherberg, Germany; 280 Allergy and Respiratory Research Group, Centre for Population Health Sciences, Medical School, The University of Edinburgh, Edinburgh, UK; 281 Child Health, Queen’s University Belfast and Royal Belfast Hospital for Sick Children, Belfast, UK; 282 Department of Thoracic Medicine, University Hospital of Heraklion, Crete, Greece; 283 University Hospital of Mont-Godinne, Catholic University of Louvain, Yvoir, Belgium; 284 Sorbonne Universités, UPMC Univ Paris 06, UMR_S 1158 Neurophysiologie Respiratoire Expérimentale et Clinique, Paris, France; 285 INSERM, UMR_S 1158 Neurophysiologie Respiratoire Expérimentale et Clinique, Paris, France; 286 Department R3S, AP-HP, Groupe, Paris, France; 287 Department of Pediatrics and Child Health, Department of Immunology, Faculty of Medicine, University of Manitoba, Winnipeg, MB Canada; 288 Sociedad Paraguaya de Alergia Asma e Inmunología, Asunción, Paraguay; 289 Julius Center of Health Sciences and Primary Care, University Medical Center Utrecht, University of Utrecht, Utrecht, The Netherlands; 290 Division of Allergy, Clinical Immunology and Rheumatology, Department of Pediatrics, Federal University of São Paulo, São Paulo, Brazil; 291 Kyrgyzstan National Centre of Cardiology and Internal Medicine, Euro-Asian Respiratory Society, Bishkek, Kyrgyzstan; 292 Pulmonary Division, Heart Institute (InCor), Hospital da Clinicas da Faculdade de Medicina da Universidade de Sao Paulo, São Paulo, Brazil; 293 Academic Medical Centre, University of Amsterdam, Amsterdam, The Netherlands; 294 Department of Epidemiology, CAPHRI School of Public Health and Primary Care, Maastricht University, Maastricht, The Netherlands; 295 Sidkkids hospitala and Institute of Health Policy, Management and Evaluation, Toronto, ON Canada; 296 Centre of Pneumology, Faculty of Medicine, University of Coimbra, Coimbra, Portugal; 297 Division of Allergy and Clinical Immunology, University of Salerno, Salerno, Italy; 298 Division of Immunopathology, Department of Pathophysiology and Allergy Research, Center for Pathophysiology, Infectiology and Immunology, Medical University of Vienna, Vienna, Austria; 299 Pneumology and Allergy Department, Hospital Clínic, Clinical and Experimental Respiratory Immunoallergy, IDIBAPS, Barcelona, Spain; 300 Department of Lung Diseases and Clinical Allergology, University of Turku, Turku, Finland; 301 Unité de Pharmacoépidémiologie, CHU-Lyon - UR 5558 CNRS, Université Claude Bernard, Lyon, Villeurbanne, France; 302 Clinical Immunology and Allergy Unit, Department of Medicine Solna, Karolinska Institutet and University Hospital, Stockholm, Sweden; 303 Department of Chest Medicine, Centre Hospitalier Universitaire Dinant-Godinne, Université Catholique de Louvain, Yvoir, Belgium; 304 FILHA, Finnish Lung Association, Helsinki, Finland; 305 Gérontopôle, CHU Toulouse, Toulouse, France; 306 Centre for Respiratory Medicine and Allergy, Institute of Inflammation and Repair, Manchester Academic Health Science Centre, The University of Manchester, Manchester, UK; 307 University Hospital of South Manchester, Manchester NHS Foundation Trust, Manchester, UK; 308 Pulmonary Unit, Department of Cardiology, Thoracic and Vascular Medicine, Arcispedale S.Maria Nuova/IRCCS, Research Hospital, Reggio Emilia, Italy; 309 Regional Agency for Health and Social Care, Reggio Emilia, Italy; 310 Division of Allergy and Immunology, Department of Pediatrics, Siriraj Hospital, Mahidol University Faculty of Medicine, Bangkok, 10700 Thailand; 311 Pulmonary Environmental Epidemiology Unit, CNR Institute of Clinical Physiology, Pisa, Via Trieste 41, 56126 Pisa, Italy; 312 CNR Institute of Biomedicine and Molecular Immunology “A. Monroy”, Via U. La Malfa 153, 90146 Palermo, Italy; 313 Department of Medicine, Pulmonary and Critical Care Medicine, University Medical Center Giessen and Marburg, Philipps-University Marburg, Marburg, Germany; 314 Sotiria Hospital, Athens, Greece; 315 Department of Otorhinolaryngology, HNO-Klinik, Universitätsklinikum Düsseldorf, Düsseldorf, Germany; 316 Hôpital Albert Calmette, CHRU, Lille, France; 317 Asthma UK, Mansell Street, London, UK; 318 Department of Otolaryngology, Yong Loo Lin School of Medicine, National University of Singapore, Singapore, Singapore; 319 Eshelman School of Pharmacy, University of North Carolina, Chapel Hill, NC USA; 320 Bradford Institute for Health Research, Bradford Royal Infirmary, Bradford, UK; 321 Department of Research, Olmsted Medical Center, Rochester, MN USA; 322 Cyprus International Institute for Environmental and Public Health in Association with Harvard School of Public Health, Cyprus University of Technology, Limassol, Cyprus; 323 Department of Pediatrics, Hospital “Archbishop Makarios III”, Nicosia, Cyprus; 324 The Allergy and Asthma Institute, Lahore, Pakistan; 325 Social Sciences, University of Southampton, Southampton, UK; 326 Department of Paediatrics and Child Health, Red Cross Children’s Hospital, and MRC Unit on Child and Adolescent Health, University of Cape Town, Cape Town, South Africa; 327 Universidad Católica de Córdoba, Córdoba, Argentina; 328 Department of Otolaryngology, Head and Neck Surgery, Beijing Tongren Hospital, Capital Medical University, Beijing, 100730 China; 329 University Clinic of Respiratory and Allergic Diseases, Golnik, Slovenia; 330 Department of Physiology, CHRU and Vice President for Research, University Montpellier, Montpellier, France; 331 EPAR UMR-S UPMC, Paris VI, Paris, France

**Keywords:** EIP on AHA, European Innovation Partnership on Active and Healthy Ageing, Chronic respiratory diseases, AIRWAYS ICPs, MACVIA, ARIA, Scaling up

## Abstract

Action Plan B3 of the European Innovation Partnership on Active and Healthy Ageing (EIP on AHA) focuses on the integrated care of chronic diseases. Area 5 (Care Pathways) was initiated using chronic respiratory diseases as a model. The chronic respiratory disease action plan includes (1) AIRWAYS integrated care pathways (ICPs), (2) the joint initiative between the Reference site MACVIA-LR (*Contre les MAladies Chroniques pour un VIeillissement Actif*) and ARIA (Allergic Rhinitis and its Impact on Asthma), (3) Commitments for Action to the European Innovation Partnership on Active and Healthy Ageing and the AIRWAYS ICPs network. It is deployed in collaboration with the World Health Organization Global Alliance against Chronic Respiratory Diseases (GARD). The European Innovation Partnership on Active and Healthy Ageing has proposed a 5-step framework for developing an individual scaling up strategy: (1) what to scale up: (1-a) databases of good practices, (1-b) assessment of viability of the scaling up of good practices, (1-c) classification of good practices for local replication and (2) how to scale up: (2-a) facilitating partnerships for scaling up, (2-b) implementation of key success factors and lessons learnt, including emerging technologies for individualised and predictive medicine. This strategy has already been applied to the chronic respiratory disease action plan of the European Innovation Partnership on Active and Healthy Ageing.

**Electronic supplementary material:**

The online version of this article (doi:10.1186/s13601-016-0116-9) contains supplementary material, which is available to authorized users.

## Background

Health and care services in Europe are undergoing changes to adapt systems to the growing demands caused by the expansion of chronic diseases and ageing. This restructuring involves the development and testing of innovative solutions as well as the implementation of the most successful pilots. The multitude of good practices developed throughout the European Union favours a comprehensive and multi-dimensional scaling-up strategy at European level [1].

The European Commission DG Santé (Directorate General for Health and Food Safety) and DG CNECT (Directorate General for Communications Networks, Content and Technology) launched the European Innovation Partnership on Active and Healthy Ageing (EIP on AHA) to enhance European Union competitiveness and to tackle societal challenges through research and innovation (Table 1) [2].

**Table 1:** Priority areas and action plans of the EIP on AHA

Priority areas	Action plans
Prevention of diseases and health promotion	
A1	Innovative ways to ensure that patients adhere to their treatment
A2	Innovative solutions for personalised health management, with focus on falls prevention
A3	Action for preventing functional decline and frailty, with a particular focus on malnutrition
Care and cure	
B3	Scaling up and replication of successful innovative integrated care models for CD amongst older patients, such as through remote monitoring
Active and independent living of older adults	
C2	Improving the uptake of interoperable independent living solutions including guidelines for business models
Horizontal topics	
D4	Networking and knowledge sharing on innovation for age-friendly environments

Chronic respiratory diseases are the pilot for chronic diseases of the EIP on AHA Action Plan B3 [3, 4]. Several effective plans exist in Europe for chronic respiratory diseases, but they are rarely deployed to other regions or countries. There is an urgent need for scaling up strategies in order to (1) avoid fragmentation, (2) improve health care delivery across Europe, (3) speed up the implementation of good practices using existing cost-effective success stories and (4) meet the triple win of the EIP on AHA:

- Enabling European citizens to lead healthy, active and independent lives while ageing.
- Improving the sustainability and efficiency of social and health care systems.
- Boosting and improving the competitiveness of the markets for innovative products and services, responding to the ageing challenge and creating new opportunities for businesses.

This paper presents the scaling up strategy for chronic respiratory diseases strictly following the five-step framework scaling up strategy of the EIP on AHA. It may be used as a model for scaling up activities in other areas of the EIP on AHA and other chronic diseases.

## AIRWAYS ICPs, the pilot for chronic diseases of the EIP on AHA

Chronic respiratory diseases include a variety of diseases such as airway diseases (allergic and non-allergic asthma, rhinitis, rhinosinusitis and COPD), occupational lung diseases, sleep apnoea syndrome, interstitial diseases, pulmonary vascular diseases and genetic diseases such as cystic fibrosis [5, 6]. Over 1 billion people in the world suffer from chronic respiratory diseases. They represent one of the priorities of the European Union (3053rd and 3131st Conclusions of the European Union Council, 2010 and 2011) [7, 8], World Health Organization (WHO 2013–2020 Noncommunicable Disease Action Plan) and the United Nations (High Level meeting on Non-Communicable Diseases, 2011) [9]. The 2011 Polish Presidency of the European Union Council made the prevention, early diagnosis and treatment of asthma and allergic diseases a priority for the European Union’s public health policy in order to reduce health inequalities [7]. The early determinants of chronic respiratory diseases were reinforced during the Cyprus Presidency of the European Union Council [10]. The 2014 Italian Presidency of the European Union Council has prioritized chronic respiratory diseases. Chronic respiratory diseases represent a model of chronic diseases due to their prevalence, burden (e.g. 3 million annual deaths due to COPD), and comorbidities with other chronic diseases [11].

The initiative AIRWAYS ICPs (Integrated care pathways for airway diseases) [3] has been approved by the EIP on AHA as the model of chronic diseases of the B3 Action Plan. It is a GARD (Global Alliance against Chronic Respiratory Diseases, World Health Organization) Research Demonstration Project [5]. It was launched by NHS England (National Health Service, Newcastle, February 2014) [12] and has been endorsed by the EIP on AHA Reference Site Network.

The objectives of AIRWAYS ICPs are to launch a collaboration to develop practical multisectoral care pathways (ICPs) to reduce chronic respiratory disease burden, mortality and multimorbidity. AIRWAYS-ICPs proposes a feasible, achievable and manageable project from science to guidelines and policies using existing networks and stakeholders committed to the Action Plan B3 of the EIP on AHA and GARD [5]. It is implemented and scaled up in Europe by the EIP on AHA and globally with GARD.

AIRWAYS-ICPs has strategic relevance to the European Union Health Strategy and the World Health Organization Noncommunicable Diseases Action Plan (2013–2020). It adds value to existing public health knowledge (Table 2).

**Table 2:** List of activities implemented by AIRWAYS ICPs

	AIRWAYS ICPs proposal	Implementation
1	Proposing a common framework of care pathways for chronic respiratory diseases to facilitate comparability and trans-national initiatives, and plans targeted to all populations according to culture, health systems and income	A repository is under development (PROEIPAHA) and the GARD strategy for adaptation to cultural beliefs and barriers is used [6]
2	Developing a strategy for low and middle-income settings	AIRWAYS ICPs uses existing WHO programmes such as the WHO GARD, WHO PEN, the essential list of drugs [5, 13, 14] and management plans already successfully tested in low and middle-income countries [13, 15, 16]
3	Aiding risk stratification in chronic disease patients with a common strategy	A common risk stratification strategy for all chronic diseases is available [17–19]
4	Defining important questions on chronic respiratory diseases in the elderly	Questions on asthma-COPD and rhinitis have been examined using a Delphi process (in preparation)
5	Developing integrated care pathways for chronic respiratory diseases and their comorbidities, with a specific focus on the elderly	Developing ICPs for chronic respiratory diseases and their comorbidities, with a specific focus on the elderly [20–25] Building a sentinel network for asthma and other allergic diseases [26]
6	Tackling chronic diseases across the life cycle	Chronic respiratory diseases occur along the life cycle and they should be prevented, diagnosed and managed early to promote AHA [7, 8, 10, 27]
7	Interacting with frailty in chronic respiratory disease (EIP on AHA Action Plan A3) and defining active and healthy ageing	Frailty is associated with chronic diseases and chronic respiratory disease. It is important to consider frailty in the management of chronic respiratory disease and to use an operational definition of AHA [28–33]
8	Implementing emerging technologies for individualised and predictive medicine in accordance with guidelines proposed by the European Commission (https://www.casym.eu)	MASK (MACVIA–ARIA Sentinel NetworK) uses emerging technologies to develop a management strategy of rhinitis and asthma multimorbidity. It is available in 15 European countries [26, 34]
9	Having a significant impact on the health of citizens in the short term (reduction of morbidity, improvement of education in children and of work in adults), the long-term (AHA), and in the development of health promotion	Asthma and COPD national plans are cost-efficient. Some have been scaled up successfully [35] New hypotheses concerning the development of allergy have been recently proposed. They may lead to novel prevention strategies [36, 37]
10	Educational activities	Educational activities are part of any scaling up strategy
11	Stratification of health systems in Europe and beyond (EIP on AHA Action Plan A3, AA4-B3)	DG Connect has initiated this project (Wouter, submitted)

## Five-step framework scaling up strategy of the EIP on AHA

Scaling up is often considered as a continuous process of change and adaptation that can take different forms [38]. The EIP on AHA has proposed a 5-step framework for developing an individual scaling up strategy. Area 5 has already used all these steps (Table 3). The scaling up process of AIRWAYS ICPs has already been initiated, during an Action Plan B3 meeting in Brussels (March 2014).

**Table 3:** The 5-step framework of EIP on AHA scaling up strategy

Step	Scaling up strategy	Individual scaling up strategy
*What to scale up*		
1	Database of good practices	
2	Assessment of viability of the scaling up of good practices	
3	Classification of good practices for local replication	
*How to scale up*		
4	Facilitating partnerships for scaling up	
5	Implementation Key success factors and lessons learnt	Planning and initiating the service
		Setting up a system for change
		Organisational process and design choices
		Training and skills for the work force
		Appropriate resourcing for equipment
		Integration of clinical record systems
		Creating capacity
		Monitoring, evaluation and dissemination

In order to achieve a successful outcome for scaling up of innovative practice, the workforce should be appropriately educated in disease management, the necessary skills (e.g. spirometry, inhaler technique) should be present, and sufficient capacity made available both for training and the extra time necessary in consultation with the individual patient. These were critical factors in achieving success in the Finnish asthma and COPD ten year plans [39]. Clinical recording systems need to be integrated to facilitate audit and appropriate sharing of clinical records.

## Application of the EIP on AHA scaling up strategy to chronic respiratory diseases

### Good practices in chronic respiratory diseases

#### AIRWAYS ICPs

Six commitments for action were submitted to the EIP on AHA to support AIRWAYS ICPs. Their good practices are complementary for the scaling up strategy (Table 4).

**Table 4:** Good practices of the EIP on AHA Commitments for Action on chronic respiratory diseases

	Activity		Expertise
MACVIA-LR (Languedoc Roussillon)	AIRWAYS ICPs Noncommunicable Diseases global approach of multimorbidity Frailty and chronic respiratory disease, a social approach MASK Eurobiomed	See Table 2	Founder of AIRWAYS ICPs Uniform definition of Noncommunicable Diseases severity and control with implementation in rural remote areas and rheumatology Definition of AHA and implementation at the social level with the French national retirement fund (CARSAT) ICT solution for rhinitis and asthma EUROBIOMED is the catalyst of the health sector in the Provence-Alpes-Côte d’Azur and Languedoc-Roussillon regions. We provide resources and initiatives to help life science companies achieve their business goals and improve life through innovations in health
Finland	Finnish asthma, COPD and allergy plans	[39–41]	Finnish plans for asthma [40], allergy [41] and COPD [39] are the prototypes of national plans for chronic respiratory diseases globally [42]
Norway	Deployment of the Finnish allergy plan to Norwegian regions	[43]	Deployment of the Finnish allergy plan to all the regions of Norway. This expertise can be used to deploy national plans to regions A European generic platform to reduce the allergy burden was created based upon the Finnish Asthma and Allergy plan
Poland	Senioral policy of Poland following the EIP on AHA recommendations including the 2011 EU Council recommendations	[7, 8] [33, 44]	The Commitment for Action of Poland was the initiator of the EU Council policy on chronic respiratory disease in children [7] and further developed the senioral policy of Poland which follows the EIP on AHA proposals. This seems to be the first AHA national project
Portugal	National coordination and national plan for all chronic respiratory diseases	[45]	The national coordination is led by the Directorate General of Health and includes all stakeholders required for a national plan which is deployed in the regions. The plan follows the Portuguese National Programme for Respiratory Diseases (PNDR)
Turkey	National coordination and Role of the chronic respiratory disease action plan on the ministerial Noncommunicable Diseases action plan	[46, 47]	The first national coordination of GARD including the Ministry of Health, WHO national office and major societies. Extremely successful programme with all public and private stakeholders of a country. Excellent example for scale up strategy

- AIRWAYS ICPs study groups exist in all but 2 European Union countries (Luxembourg, Malta). They follow the GARD model deployed in Turkey [46, 47] and Italy [13, 48].
- Governments of countries (e.g. Lithuania, Poland, Portugal, Turkey) or regions (e.g. Emilia-Romagna) are involved in AIRWAYS ICPs. One of the commitments for action (Norway) is a joint action between the Ministry of Health of Finland and Norway [43].

#### Other international, national or regional projects

Many guidelines, ICPs and national plans exist for the most common chronic respiratory diseases (asthma, COPD, rhinitis).

- The Finnish plans for asthma [40], allergy [41] and COPD [39], considered to be the prototypes of national plans for chronic respiratory diseases [42]. Polastma (Poland) is, in particular, derived from the asthma plan [35]. A review on the European asthma plans based on the Finnish Asthma Plan is available [42].
- The Portuguese National Programme for Respiratory Diseases (PNDR), the first national programme including all respiratory diseases [45].
- In the Netherlands, the SMART-formulated collaborative National Action programme against Chronic Lung Diseases (NACL) aims to improve the cost-effectiveness of respiratory prescribing, while reducing hospitalisation days, productivity loss, adolescent smoking, and mortality due to asthma and COPD. Both the Ministry of Health and the collective Health Insurers Netherlands are funding the programme [13].
- Several national or regional plans on asthma, COPD, other chronic respiratory diseases and allergy.
- Guidelines or strategies for asthma [49–52], COPD [53], rhinitis [21], rhinosinusitis [54] or severe asthma [55] (Table 5).

**Table 5:** An example of scaling up strategy: ARIA (Allergic Rhinitis and its Impact on Asthma) [21, 26]

Allergic rhinitis is one of the most prevalent diseases in the world (25 % of the European Union population). Although symptoms of rhinitis appear to be trivial, the disease affects social activities as well as school and work performance [56]. It is often associated with or precedes asthma (including in the elderly) [57, 58]. Allergic rhinitis has been considered to alter AHA if not appropriately managed [7, 8]
ARIA, a guideline for allergic rhinitis and its multimorbidity with asthma, is the first multimorbidity guideline in chronic diseases. It was developed in the early 2000s in collaboration with the World Health Organization using the recommended methodology for guidelines (Shekelle) [59]. It was updated in 2008 [60]
It has been revised using the GRADE methodology (2010) [22, 61, 62]
It is the most widely used guideline for rhinitis, and for rhinitis and asthma multimorbidity globally [21]
The ARIA classification of allergic rhinitis severity has been used for the development of Health Technology Assessment guidelines, in particular in the US [63]
ARIA recommendations have been adopted by government guidelines (Brazil, Portugal, Singapore)
ARIA is implemented in 64 countries and the pocket guide of the guideline has been translated into 52 languages
MASK-rhinitis (MACVIA–ARIA Sentinel NetworK for allergic rhinitis) is a care pathway centred around the use of Information and Communications Technology (ICT) tools and a clinical decision support system (CDSS) based on ARIA [26, 34]. This tool can be used by older adults
Over 600 scientific papers have used ARIA for the classification of allergic rhinitis in clinical practice, clinical trials, as well as epidemiologic (from pre-school children to the elderly [58]), basic and translational research [21]
- Care pathways provided by national institutions (e.g. NICE in the UK, National Institute for Health and Care Excellence or the Haute Autorité de Santé in France, ICP for acute asthma in children in Northern Ireland).
- The World Health Organization guidelines for asthma and COPD in low-income settings (WHO PEN) [14].
- Management plans already successfully tested in low and middle-income countries [15].
- A common approach to severe asthma and allergic diseases [17, 19].
- In Spain, Polibienestar Research Institute is developing a Multi-Agent Simulator for people requiring prolonged mechanical ventilation based on the validated LTCMAS [64] and following the Canadian model [65], which is easily replicable and transferrable to other healthcare systems and to other diseases. Moreover, this tool offers great possibilities for scaling-up and for supporting the decision-making process of health professionals and policy-makers.
- Multimorbidity guidelines for chronic respiratory diseases do not exist, except for rhinitis and asthma [21].
- The risk for developing a COPD has only been studied in Italy and represents a chart risk applicable to the entire Europe.
- Palliative approaches to care in chronic respiratory disease, and planning end-of-life decisions and care/advanced care.
- Guidelines with a specific target on old age adults do not exist. A Delphi process is ongoing.

#### Guidance documents for primary care

Some guidance documents are specifically directed to primary care—where most patients with chronic respiratory diseases are managed—such as COPD-Australia (Lung Foundation Australia with Thoracic Society of Australia and New Zealand) and Asthma Management in Australia (National Asthma Council Australia). IPCRG (International Primary Care Respiratory Group) has undertaken a mapping on national guidelines used by primary care for COPD, asthma, rhinitis, obstructive sleep apnea and stop smoking (https://www.theipcrg.org/display/ResMapping).

### Database

A centralized repository of evidence is developed to preserve data throughout the lifecycle of the project. The repository is under development by the Commission.

### Assessment of viability of the scaling up of good practices

The members of AIRWAYS ICPs, ARIA and GARD [6, 13, 48] have experience in working together and have already scaled up several chronic respiratory disease good practices. Scaling up for ARIA and GARD follows the 7 key characteristics of the CORRECT features: Credible, Observable, Relevant, Relative advantage, Easy and Compatible [66, 67]. The success of the scaling up strategy and its long-term viability (over 15 years for ARIA and 8 years in GARD) has been demonstrated. GARD has been scaled up in several countries at governmental levels [13, 46–48].

Members of 13 EIP on AHA Reference Sites have agreed on the AIRWAYS ICPs concept and are co-authors of the paper [3]. A meeting of all EIP on AHA Reference Sites was co-organised by the Région LR, North England and the EIP on AHA Reference Site Collaborative Network to scale up AIRWAYS ICPs in all Reference Sites (October 21, 2014).

The viability of ARIA and GARD has been demonstrated. The viability of AIRWAYS ICPs will be analysed according to the set of parameters provided by the Commission in the near future. The analysis will be carried out within 6 months by an AIRWAYS ICPs expert panel and revised by an independent expert panel (6 additional months). The meeting for the analysis of the viability took place in Lisbon (Directorate General of Health of Portugal), July 1-2, 2015 in collaboration with the World Health Organization GARD [68].

### Classification of good practices for replication

Feasibility has been reviewed for the Finnish Asthma Plan (Table 6). It is expected that AIRWAYS ICPs following the expertise raised in ARIA and GARD will have a similar feasibility.

**Table 6:** Classification of good practices for replication: the example of the Finnish Asthma Plan [40]

Items		Example of the Finnish Asthma Plan
Knowledge—gaps	Between knowledge and practice (research, specific)	The plan has been [69] tested and validated at the national level [40]
	Existence of tested solutions (good examples, specific)	It has shown cost-effective reduction of hospitalisations, deaths and disability
	Large variations between countries (good examples, general)	The Finnish Asthma Plan has been deployed successfully to over 25 countries globally including developing countries. The same effectiveness has been demonstrated [70, 71]. The Finnish Asthma Plan is considered to be the model of all asthma plans in the world [35]
Reaction time	Calendar (time needed for implementation	The Finnish Asthma Plan was a 10-year plan. Most indicators were found to change significantly after 24–36 months, but the effectiveness improved over the 10-year programme. In Brazil, an impact at population morbidity indicators was found after 24 months
	Effects/visibility (time needed to assess impact)	
Stewardship	Administrative and political capacity. Leadership, inside the health sector and in other sectors (Health in All Policies)	Many plans are national plans supported by the Ministry of Health or the department of health of the region (e.g. Minas Gerais, Brazil). All stakeholders including health (specialists, GPs, nurses, pharmacists, other health care professionals) and social carers as well as patients are involved in the plan. A specific action is devoted to education, coaching and training
Political agenda	Electoral programme	
	Social concerns	A specific attention has been put on social concerns and a promotion in the country at all levels (citizens and patients, health and social carers, politicians) has been continuously monitored
	Crisis	
	International institutions recommendations/conditions	The Finnish Asthma Plan and its follow up (the Finnish Allergy Programme) [41, 72] has been endorsed by the Finnish Ministry of Health. Some plans in developed and developing countries (globally) are also under the Ministry of Health leadership and some have been endorsed by WHO GARD (GARD demonstration project). The Finnish Asthma Plan is listed in asthma guidelines
Costs and affordability	It is important to consider the cost of the programme for selecting priority areas for investment. Certain decisions could need relevant investments (e.g. equipment, personnel, etc.) while others involve low direct economic cost (e.g. anti-tobacco strategies and legislation). The costs of a programme have to be considered in the context of the economic situation of the country (GDP/inhabitant; expansion/recession/stagnation; private and public debt; etc.)	The Finnish Asthma Plan is comprehensive and includes treatments, preventive measures (e.g. tobacco smoking), action plans, education at all levels. It was found to be cost-effective. This has been demonstrated in Finland, but also in other countries such as Brazil [42, 73, 74]. Thus, reducing the asthma burden is cost-effective in countries with different GDP/inhabitant, health and economic systems
Acceptability	The support or the opposition that a certain policy is going to attract	The Plan was extremely well accepted in all countries where it was promoted [42]
Monitoring capability	The availability of the necessary information to monitor the starting point, the processes and the outcomes	Baseline information on the burden of asthma is available even though in most developing countries there is no information [75]. Information on the success of the programme was easily documented [35, 70, 71] and carefully monitored
	It highlights also the importance of transparency	National (or regional) statistics are transparent
Contextual factors	Demographics	The Finnish Asthma Plan was a national plan covering the entire country. Some plans are regional plans (Bahia or Minais Gerais)
	Social and economic conditions	The Finnish Asthma Plan targeted the entire country. The Minais Gerais plan targets children in deprived areas (“favelas”) who are at high risk of severe exacerbations and death [76] as does the severe asthma programme established in Bahia, dealing with children and adults [70]
	Cultural factors	In Finland, barriers are not very important. However, in many developing countries, cultural barriers have been carefully considered according to a WHO report [6]. They include culture, gender issues, socio-economic inequalities, health care access, access to essential medications and techniques
	Other non-health care determinants of health that impact on population health and wellbeing	

### Facilitating partnership for scaling up

#### Collaborator’s role

The ARIA programme includes over 300 members and AIRWAYS ICPs includes 445 members. The paper describing the AIRWAYS ICPs proposal is co-authored by 250 members (all stakeholders: health care professionals, social carers, patients, government officers, methodologists, etc.) [3]. All of the members are very committed to the implementation of AIRWAYS ICPs. National and regional groups have been initiated in all but 2 European Union countries. In countries where health care is regionalised [59], many regional groups are in place.

#### Role of scientific societies

AIRWAYS ICPs is in line with the mission and vision of scientific societies which aim to (1) promote research, (2) collect, assess and diffuse scientific information, (3) represent a scientific reference body for other scientific, health and political organisations and an advocate towards political organisation and the general public, (4) encourage and provide training, continuous education and professional development and (5) collaborate with patients and lay organisations in the area of their field in order to lead the way towards better understanding, prevention, management and eventual cure of diseases. The European Academy of Allergy and Clinical Immunology (EAACI), the European Respiratory Society (ERS), the European Rhinology Society (ERS), the European Union Geriatric Medicine Society (EUGMS), the International Academy of Pediatrics and the International Primary Care Respiratory Group (IPCRG) are the major societies in Europe of their respective field and are all members of AIRWAYS ICPs. A recent meeting on precision medicine in airways and allergic diseases was held at the European Union Parliament with these societies [77, 78]. The activities of IPCRG are summarized in Additional file 1.

#### Role of patient’s organisations

The goal and rationale of patient involvement in medical decisions is patient empowerment. Empowered patients know their disease. Patient empowerment commences with the initial consultations at the primary care level encompassing discussions about the patient’s ideas, concerns and expectations coupled with patient education about the specific disease process, what can be done to ameliorate the disease and ultimately self-management. Patients have the skills and motivation to take good care in their everyday life, to adjust their treatment, and are prepared for new or potentially exacerbating situations. They are able to detect side-effects, contact healthcare professionals when necessary and they adhere to the treatment regime. Many tools support empowerment, shared decision making models and patient education. Patient empowerment should be included in the health care professional’s curriculum. For an optimal dissemination of good practices, there is a need for patient involvement and empowerment.

There are recommendations to secure patient organization/patient involvement at national (e.g. The Netherlands ZonMW) and also at European Union level [79, 80].

EFA (European Federation of Allergy and airways diseases patient’s association), the major patient’s organisation for respiratory and allergic diseases in Europe, has been very active for AIRWAYS ICPs [77, 78].

#### Diffusion of good practices

All European Union countries should be included.

The European Geriatric Medicine, the official organ of the European Union Geriatric Medicine Society (EUGMS), has initiated a column of the EIP on AHA to publish important activities of the EIP on AHA in order to inform the medical community [2]. Several papers have already been published [2, 29, 44, 81–85].

- *Reference Site Network*: The Reference Site Network is already committed to AIRWAYS-ICPs (decision taken during the Montpellier meeting).
- *Action Groups*: Area 5 of Action Group B3 is leading AIRWAYS ICPs.
- *Event and dedicated scaling up/twinning sessions*: Several events have already taken place (Table 7).

**Table 7:** AIRWAYS ICPs 2014 events

Date	Location	Event and goals
27-02	Newcastle (UK)	Launch of AIRWAYS ICPs by Dr. M Bewick, Deputy National Medical Director of NHS England, [12]
12-05	Athens (Greece)	AIRWAYS ICPs was presented to the EIP on AHA
09-06	Copenhagen (Denmark)	European Academy of Allergy and Clinical Immunology (EAACI). A symposium was organized (1000 participants) and a working meeting held immediately after: AIRWAYS ICPs and MACVIA–ARIA [26]
17-08	Bahia (Brazil)	WHO GARD annual meeting. Presentation of AIRWAYS ICPs and MACVIA–ARIA to the GARD members and WHO. Acceptance of AIRWAYS ICPs to strengthen the 2013–2020 Noncommunicable Diseases WHO Action Plan [86, 87]
16-09	Rotterdam (NL)	Annual meeting of the European Union Geriatric Medicine Society (EUGMS): Presidential lecture on AIRWAYS (T Strandberg, President of the Society)
09-10	Dubrovnik (Croatia)	Annual meeting of the Croatian Respiratory Society. AIRWAYS ICPs and MACVIA–ARIA were presented (M Niculinic, President of the Society)
16-10	Rome (Italy)	The Italian Presidency of the European Union Council has made chronic respiratory diseases one of the priorities. A GARD Italy meeting was held at the Ministry of Health. AIRWAYS ICPs was presented among other projects to be included in the Priority
20-10	Montpellier (France)	The Region Languedoc Roussillon (in collaboration with the region North England and the EIP on AHA Reference Site Collaborative Network) invited one member from each Reference Site to scale up AIRWAYS ICPs. The Collaborative Network decided to include AIRWAYS ICPs in its priorities for scaling up and implementation (M Bewick, R Pengelly, Secretary of State of Northern Ireland) [28, 29]
05-11	Salzburg (Austria)	Annual meeting of the Austrian Allergy Society
07-11	Guangzhou (China)	Annual meeting: Discussion for the deployment of AIRWAYS ICPs and MACVIA–ARIA in China (NS Zhong, former President of the Chinese Medical Association) [88]
20-11	Oslo (Norway)	Commitments for Action Oslo, Helsinki and Montpellier (K Lodrup Carlsen, T Haahtela, JB). The agreement for the deployment of the Finnish Allergy Programme in Norway was discussed at the Ministry of Health [43]
- *Network of excellence centres in respiratory and allergic diseases*: It includes the Commitments for Action (EIP on AHA action Plan B3), Reference Sites of the EIP AHA, the Global Allergy and Asthma European Network (GA^2^LEN) and members of AIRWAYS ICPs. GA^2^LEN, a Sixth European Union Framework Programme for Research and Technological Development (FP6) Network of Excellence, was created in 2005 as a vehicle to ensure excellence in research bringing together research and clinical institutions to combat fragmentation in the European research area and to tackle allergy in its globality [89]. The GA^2^LEN network has benefited greatly from the voluntary efforts of researchers who are strongly committed to this model of pan-European collaboration. The network was organized in order to increase networking for scientific and clinical projects in allergy and asthma around Europe.

### Implementation, key success factors and lessons learnt

#### Planning and initiating the service

- *Needs for AIRWAYS ICPs*, in particular in elderly adults and co-morbid diseases, are clear. AIRWAYS ICPs was developed following the research priorities set by the World Health Organization on chronic respiratory diseases [90].
- *The strategy, the road map and the first implementation results* have been published [4].
- *ICPs for asthma have been shown to be highly cost*-*effective in* different settings [15, 35]. Studies in developed and developing countries have shown a cost-effective reduction of hospitalisations and mortality.

#### Setting up a system for change

- *Good understanding*: The members of ARIA, GARD and AIRWAYS ICPs have perceived the need for innovation, and consider it beneficial and congruent with central ideas and concepts. Deployment has been made to all stakeholders including patients and citizens. The results of the ARIA and GARD initiatives are clear [13, 46, 91–98]. Since the same methodology is used for AIRWAYS ICPs enhanced by the EIP on AHA scaling up strategy, there is no reason for a lack of understanding. The present paper is co-authored by over 450 authors from 72 countries in order to enhance understanding for different cultures, settings, health systems and languages.
- *Implementation of emerging technologies for predictive and personalised medicine*. Systems medicine is an emerging discipline [18, 77, 78, 99]. It combines high-throughput analyses of all human genes and their products with computational, functional and clinical studies. The aim is to gain detailed understanding of disease mechanisms, and how they vary between different patient groups. This understanding can be exploited for predictive and personalised medicine, according to guidelines proposed by the European Commission (https://www.casym.eu). The first implementations may reach the clinic within the next five years for serious diseases that require costly treatments [100].
- *Political endorsement*: Several meetings have been organised by the European Union. In particular, the Polish Priority of the Council [7, 8] which “WELCOMES existing networks and alliances, such as the Global Allergy and Asthma European Network (GA^2^LEN) and Global Alliance against Respiratory Diseases (GARD)”. There are recommendation: (i) to give appropriate consideration to prevention, early diagnosis and treatment, (ii) to strengthen cooperation with relevant stakeholders, (iii)to exchange best practices, (iv) to support national centres and existing international research networks (v) to find cost-effective procedures by using health technology assessment, (vi) to improve health care system standards relating to chronic respiratory diseases, (vii) to consider the use of e-Health tools and innovative technologies for prevention, early diagnosis and treatment of chronic respiratory diseases, and finally (viii) to support Member States by the “Commission developing and implementing effective policies, improving networking among institutions responsible for the implementation of programmes.”

A meeting at the European Union Parliament under the leadership of the Cyprus Presidency of the European Union Council [10] and a GARD meeting at the Italian Ministry of Health during the Presidency of the Council both reinforced the importance of chronic respiratory diseases for their early detection and management to improve AHA. The present document was presented at a meeting in Lisbon, Portugal (July 1–2, 2015) organised by the Reference Site Network of the EIP on AHA in collaboration with European Union regions and the Directorate General of Health.

MACVIA-LR is supported by a strong political endorsement at the regional level. ARIA has been adopted by several governmental policies. AIRWAYS ICPs has been launched in collaboration with NHS England, Scotland, Northern Ireland, the Ministry of Health of Portugal, Poland and Lithuania and several governments of regions (e.g. Emilia Romagna, Basque Country).

- *Engagement of relevant stakeholders*: In ARIA, GARD and AIRWAYS ICPs, all relevant stakeholders have been included and are highly motivated: health care professionals (physicians, pharmacists, nurses, physiotherapists and others), social workers, policy makers. A special effort has been made for patient empowerment. A European Union Parliament session led by EFA, the largest European patients’ organisation in asthma and airway diseases, has been organised in collaboration with MeDALL (Mechanisms of the Development of Allergy, FP7 project) [36, 37], in May 2015. Professional societies and groups should be enlisted as active collaborators in order to enhance and even drive uptake at the country level.
- *Financial viability and business model*: It has been shown that the implementation of the Finnish national plans, ARIA and GARD does not require large resources. However, AIRWAYS ICPs will require arrangements for the reimbursement of the services.

#### Organisational process and design choices

- *Investing in human capital*: Training and reskilling the work force is an essential and fundamental component of AIRWAYS ICPs. This may require initial and continuing investment to ensure that the workforce possesses the appropriate knowledge, skills and equipment to fulfil its roles, as show by some very successful ARIA and GARD initiatives. AIRWAYS ICPs should shall go a step further, however, and be fully implemented countrywide. The EIP on AHA Reference Site Network has offered its help. The present paper has been co-authored by many professional leaders from over 70 countries to build a global momentum.
- *Integrating ICT solutions*: Telemedicine represents a possible specific advanced tool of ICT in chronic respiratory disease management and secondary prevention. ICT solutions are integrated to support AIRWAYS ICPs implementation and the MACVIA–ARIA Sentinel NetworK has been launched in Copenhagen (June 9, 2014). A clinical decision support system (CDSS) is being built and should be available at the end of the year. This system may form the prototype for a more complex one for asthma, COPD, other chronic respiratory diseases and co-morbidities.
- *Organisational changes*: Currently under discussion but will require flexibility in order to adapt to the needs of different areas.

#### Monitoring, evaluation and dissemination

These activities have been initiated by ARIA and GARD at the international level, but they are also part of the national and regional plans for chronic respiratory diseases. The Area 5 programme on chronic respiratory diseases will benefit from previous expertise, successes and failures to propose refined and updated activities.

- *Assessment indicators*: In asthma and COPD, hospitalisation rates and mortality are two indicators of interest and are responsive to change within 2–3 years. In rhinitis, these indicators cannot be used. Control is applicable to asthma, COPD and/or rhinitis and quality of life is applicable to all 3 diseases. An economic evaluation was found to be effective in asthma in many countries [40, 74].
- *Mutual learning*: Learning Networks for learning and sharing best practices are in place for chronic respiratory diseases. Scientific societies and patient’s organisations are of importance in the process.
- *Dissemination activities*: One of the strengths of ARIA and GARD, and also already AIRWAYS ICPs, is the great ability to disseminate information and guidelines in countries of the European Union and globally.
- *Scaling up of the new good practices*: Another strength of ARIA and GARD is the capacity to scale up good practices in countries of the European Union and elsewhere.

## Conclusions

The scaling up strategy of AIRWAYS ICPs confirms that the proposed strategy of the EIP on AHA is simple and easy to follow. It may be used as a model for the scaling up strategies of other projects of the EIP on AHA.

## Additional file

10.1186/s13601-016-0116-9 IPCRG scaling up activities.

## Abbreviations

AIRWAYS ICPs: integrated care pathways for airway diseases
ARIA: Allergic Rhinitis and its Impact on Asthma
COPD: chronic obstructive pulmonary disease
DG: Directorate General
EIP on AHA: European Innovation Partnership on Active and Healthy Ageing
GA^2^LEN: Global Allergy and Asthma European Network (FP6)
GARD: WHO Global Alliance against Chronic Respiratory Diseases
ICP: integrated care pathway
IPCRG: International Primary Care Respiratory Group
MACVIA-LR: Contre les MAladies Chroniques pour un VIeillissement Actif (Fighting chronic diseases for active and healthy ageing)
MASK: MACVIA–ARIA Sentinel NetworK
NHS: National Health Service
WHO: World Health Organization
VAS: visual analogue scale
