# Erratum to: Scaling up strategies of the chronic respiratory disease programme of the European Innovation Partnership on Active and Healthy Ageing (Action Plan B3: Area 5)

**DOI:** 10.1186/s13601-016-0135-6

**Published:** 2017-02-20

**Authors:** J. Bousquet, J. Farrell, G. Crooks, P. Hellings, E. H. Bel, M. Bewick, N. H. Chavannes, J. Correia de Sousa, A. A. Cruz, T. Haahtela, G. Joos, N. Khaltaev, J. Malva, A. Muraro, M. Nogues, S. Palkonen, S. Pedersen, C. Robalo-Cordeiro, B. Samolinski, T. Strandberg, A. Valiulis, A. Yorgancioglu, T. Zuberbier, A. Bedbrook, W. Aberer, M. Adachi, A. Agusti, C. A. Akdis, M. Akdis, J. Ankri, A. Alonso, I. Annesi-Maesano, I. J. Ansotegui, J. M. Anto, S. Arnavielhe, H. Arshad, C. Bai, I. Baiardini, C. Bachert, A. K. Baigenzhin, C. Barbara, E. D. Bateman, B. Beghé, A. Ben Kheder, K. S. Bennoor, M. Benson, K. C. Bergmann, T. Bieber, C. Bindslev-Jensen, L. Bjermer, H. Blain, F. Blasi, A. L. Boner, M. Bonini, S. Bonini, S. Bosnic-Anticevitch, L. P. Boulet, R. Bourret, P. J. Bousquet, F. Braido, A. H. Briggs, C. E. Brightling, J. Brozek, R. Buhl, P. G. Burney, A. Bush, F. Caballero-Fonseca, D. Caimmi, M. A. Calderon, P. M. Calverley, P. A. M. Camargos, G. W. Canonica, T. Camuzat, K. H. Carlsen, W. Carr, A. Carriazo, T. Casale, A. M. Cepeda Sarabia, L. Chatzi, Y. Z. Chen, R. Chiron, E. Chkhartishvili, A. G. Chuchalin, K. F. Chung, G. Ciprandi, I. Cirule, L. Cox, D. J. Costa, A. Custovic, R. Dahl, S. E. Dahlen, U. Darsow, G. De Carlo, F. De Blay, T. Dedeu, D. Deleanu, E. De Manuel Keenoy, P. Demoly, J. A. Denburg, P. Devillier, A. Didier, A. T. Dinh-Xuan, R. Djukanovic, D. Dokic, H. Douagui, G. Dray, R. Dubakiene, S. R. Durham, M. S. Dykewicz, Y. El-Gamal, R. Emuzyte, L. M. Fabbri, M. Fletcher, A. Fiocchi, A. Fink Wagner, J. Fonseca, W. J. Fokkens, F. Forastiere, P. Frith, M. Gaga, A. Gamkrelidze, J. Garces, J. Garcia-Aymerich, B. Gemicioğlu, J. E. Gereda, S. González Diaz, M. Gotua, I. Grisle, L. Grouse, Z. Gutter, M. A. Guzmán, L. G. Heaney, B. Hellquist-Dahl, D. Henderson, A. Hendry, J. Heinrich, D. Heve, F. Horak, J. O’. B. Hourihane, P. Howarth, M. Humbert, M. E. Hyland, M. Illario, J. C. Ivancevich, J. R. Jardim, E. J. Jares, C. Jeandel, C. Jenkins, S. L. Johnston, O. Jonquet, K. Julge, K. S. Jung, J. Just, I. Kaidashev, M. R. Khaitov, O. Kalayci, A. F. Kalyoncu, T. Keil, P. K. Keith, L. Klimek, B. Koffi N’Goran, V. Kolek, G. H. Koppelman, M. L. Kowalski, I. Kull, P. Kuna, V. Kvedariene, B. Lambrecht, S. Lau, D. Larenas-Linnemann, D. Laune, L. T. T. Le, P. Lieberman, B. Lipworth, J. Li, K. Lodrup Carlsen, R. Louis, W. MacNee, Y. Magard, A. Magnan, B. Mahboub, A. Mair, I. Majer, M. J. Makela, P. Manning, S. Mara, G. D. Marshall, M. R. Masjedi, P. Matignon, M. Maurer, S. Mavale-Manuel, E. Melén, E. Melo-Gomes, E. O. Meltzer, A. Menzies-Gow, H. Merk, J. P. Michel, N. Miculinic, F. Mihaltan, B. Milenkovic, G. M. Y. Mohammad, M. Molimard, I. Momas, A. Montilla-Santana, M. Morais-Almeida, M. Morgan, R. Mösges, J. Mullol, S. Nafti, L. Namazova-Baranova, R. Naclerio, A. Neou, H. Neffen, K. Nekam, B. Niggemann, G. Ninot, T. D. Nyembue, R. E. O’Hehir, K. Ohta, Y. Okamoto, K. Okubo, S. Ouedraogo, P. Paggiaro, I. Pali-Schöll, P. Panzner, N. Papadopoulos, A. Papi, H. S. Park, G. Passalacqua, I. Pavord, R. Pawankar, R. Pengelly, O. Pfaar, R. Picard, B. Pigearias, I. Pin, D. Plavec, D. Poethig, W. Pohl, T. A. Popov, F. Portejoie, P. Potter, D. Postma, D. Price, K. F. Rabe, F. Raciborski, F. Radier Pontal, S. Repka-Ramirez, S. Reitamo, S. Rennard, F. Rodenas, J. Roberts, J. Roca, L. Rodriguez Mañas, C. Rolland, M. Roman Rodriguez, A. Romano, J. Rosado-Pinto, N. Rosario, L. Rosenwasser, M. Rottem, D. Ryan, M. Sanchez-Borges, G. K. Scadding, H. J. Schunemann, E. Serrano, P. Schmid-Grendelmeier, H. Schulz, A. Sheikh, M. Shields, N. Siafakas, Y. Sibille, T. Similowski, F. E. R. Simons, J. C. Sisul, I. Skrindo, H. A. Smit, D. Solé, T. Sooronbaev, O. Spranger, R. Stelmach, P. J. Sterk, J. Sunyer, C. Thijs, T. To, A. Todo-Bom, M. Triggiani, R. Valenta, A. L. Valero, E. Valia, E. Valovirta, E. Van Ganse, M. van Hage, O. Vandenplas, T. Vasankari, B. Vellas, J. Vestbo, G. Vezzani, P. Vichyanond, G. Viegi, C. Vogelmeier, T. Vontetsianos, M. Wagenmann, B. Wallaert, S. Walker, D. Y. Wang, U. Wahn, M. Wickman, D. M. Williams, S. Williams, J. Wright, B. P. Yawn, P. K. Yiallouros, O. M. Yusuf, A. Zaidi, H. J. Zar, M. E. Zernotti, L. Zhang, N. Zhong, M. Zidarn, J. Mercier

**Affiliations:** 1 CHRU, University Hospital, 371 Avenue du Doyen Gaston Giraud, 34295 Montpellier Cedex 5, France; 2 MACVIA-LR, Contre les MAladies Chroniques pour un VIeillissement Actif en Languedoc Roussilon, European Innovation Partnership on Active and Healthy Ageing Reference Site, Montpellier, France; 3 INSERM, VIMA: Ageing and Chronic Diseases, Epidemiological and Public Health Approaches, U1168, Paris, France; 4 UVSQ, UMR-S 1168, Université Versailles St-Quentin-en-Yvelines, Paris, France; 5 Department of Health, Social Services and Public Safety, Belfast, Northern Ireland, UK; 6 EIP on AHA, European Innovation Partnership on Active and Healthy Ageing, Reference Site, Scottish Centre for Telehealth and Telecare, NHS 24, Glasgow, UK; 7 Laboratory of Clinical Immunology, Department of Microbiology and Immunology, KU Leuven, Louvain, Belgium; 8 European Academy of Allergy and Clinical Immunology, Zurich, Switzerland; 9 Department of Respiratory Medicine, Academic Medical Center (AMC), University of Amsterdam, Amsterdam, The Netherlands; 10 European Respiratory Society, Lausanne, Switzerland; 11 iQ4U Consultants Ltd, London, UK; 12 Department of Public Health and Primary Care, Leiden University Medical Center, Leiden, The Netherlands; 13 Global Alliance Against Chronic Respiratory Diseases (GARD), Cape Town, South Africa; 14 International Primary Care Respiratory Group, Westhill, UK; 15 Life and Health Sciences Research Institute, ICVS, School of Health Sciences, University of Minho, Braga, Portugal; 16 ProAR – Nucleo de Excelencia em Asma, Federal University of Bahia, Bahia, Brazil; 17 GARD Executive Committee, Bahia, Brazil; 18 EIP on AHA Commitment for Action, Lisbon, Portugal; 19 Skin and Allergy Hospital, Helsinki University Hospital, Helsinki, Finland; 20 Department of Respiratory Medicine, Ghent University Hospital, Ghent, Belgium; 21 Faculty of Medicine, University of Coimbra, Coimbra, Portugal; 22 Ageing@Coimbra Reference Site, Coimbra, Portugal; 23 Food Allergy Referral Centre Veneto Region, Department of Women and Child Health, Padua General University Hospital, Padua, Italy; 24 Caisse Assurance Retraite et Santé Au Travail Languedoc-Roussillon (CARSAT-LR), 34000 Montpellier, France; 25 EFA European Federation of Allergy and Airways Diseases Patients’ Associations, Brussels, Belgium; 26 University of Southern Denmark, Kolding, Denmark; 27 Centre of Pneumology, Coimbra University Hospital, Coimbra, Portugal; 28 Department of Prevention of Environmental Hazards and Allergology, Medical University of Warsaw, Warsaw, Poland; 29 Helsinki University, Helsinki University Hospital, Helsinki, Finland; 30 Center for Life Course Health Research, University of Oulu, Oulu, Finland; 31 European Union GeriatricMedicine Society, EUGMS, Oslo, Norway; 32 Center of Quality of Life Research, Vilnius University Clinic of Children’s Diseases, Vilnius University Public Health Institute, Vilnius, Lithuania; 33 European Association of Pediatrics (EAP/UEMS-SP), Brussels, Belgium; 34 Department of Pulmonology, Celal Bayar University, Manisa, Turkey; 35 Turkish Thoracic Society, Antalya, Turkey; 36 Allergy-Centre-Charité at the Department of Dermatology, Charité - Universitätsmedizin Berlin, Berlin, Germany; 37 Secretary General of the Global Allergy and Asthma European Network (GA²LEN), Berlin, Germany; 38 Department of Dermatology, Medical University of Graz, Graz, Austria; 39 Department of Clinical Research Center, International University of Health and Welfare/Sanno Hospital, Tokyo, Japan; 40 Thorax Institute, Hospital Clinic, IDIBAPS, University of Barcelona, Barcelona, Spain; 41 CIBER Enfermedades Respiratorias, Barcelona, Spain; 42 Swiss Institute of Allergy and Asthma Research (SIAF), University of Zurich, Davos, Switzerland; 43 EPAR U707 INSERM, Paris, France; 44 Department of Allergy and Immunology, Hospital Quirón Bizkaia, Erandio, Spain; 45 Centre for Research in Environmental Epidemiology (CREAL), Barcelona, Spain; 46 Hospital del Mar Research Institute (IMIM), Barcelona, Spain; 47 CIBER Epidemiología y Salud Pública (CIBERESP), Barcelona, Spain; 48 Department of Experimental and Health Sciences, University of Pompeu Fabra (UPF), Barcelona, Spain; 49 Digi Health, Montpellier, France; 50 David Hide Asthma and Allergy Research Centre, Isle of Wight, UK; 51 Shanghai Respiratory Research Institute, Vice President of Respiratory Society, Chinese Medical Association, China and Chinese Alliance Against Lung Cancer, Shanghai, China; 52 Allergy and Respiratory Diseases Clinic, DIMI, IRCCS AOU San Martino-IST, University of Genoa, Genoa, Italy; 53 Upper Airways Research Laboratory, ENT Department, Ghent University Hospital, Ghent, Belgium; 54 EuroAsian Respiratory Society, Astana City, Kazakhstan; 55 Faculdade de Medicina de Lisboa, Portuguese National Programme for Respiratory Diseases (PNDR), Lisbon, Portugal; 56 Department of Medicine, University of Cape Town, Cape Town, South Africa; 57 Section of Respiratory Disease, Department of Oncology, Haematology and Respiratory Diseases, University of Modena and Reggio Emilia, Modena, Italy; 58 Service de pneumologie IV, hôpital Abderrahman Mami, Ariana, 2080 Tunis, Tunisia; 59 Department of Respiratory Medicine, National Institute of Diseases of the Chest and Hospital, Dhaka, Bangladesh; 60 Centre for Individualized Medicine, Department of Pediatrics, Faculty of Medicine, LInköping University, Linköping, Sweden; 61 Department of Dermatology and Allergy, Rheinische Friedrich-Wilhelms-University Bonn, Bonn, Germany; 62 Department of Dermatology and Allergy Centre, Odense University Hospital, Odense, Denmark; 63 Department of Respiratory Medicine and Allergology, University Hospital, Lund, Sweden; 64 Department of Geriatrics, Montpellier University Hospital, Montpellier, France; 65 EA 2991, Euromov, University Montpellier, Montpellier, France; 66 Department of Pathophysiology and Transplantation, IRCCS Fondazione Ca’Granda Ospedale Maggiore Policlinico, University of Milan, Via F. Sforza 35, Milan, Italy; 67 Pediatric Department, University of Verona Hospital, Verona, Italy; 68 Department of Public Health and Infectious Diseases, Sapienza University of Rome, Rome, Italy; 69 Second University of Naples and Institute of Translational Medicine, Italian National Research Council, Naples, Italy; 70 Woolcock Institute of Medical Research, University of Sydney and Sydney Local Health District, Glebe, NSW Australia; 71 Quebec Heart and Lung Institute, Laval University, Québec City, QC Canada; 72 Directeur Général Adjoint, Montpellier University Hospital, Montpellier, France; 73 Health Economics and Health Technology Assessment, Institute of Health and Wellbeing, University of Glasgow, Glasgow, UK; 74 Respiratory Biomedical Unit, Institute of Lung Health, University Hospitals of Leicester NHS Trust, Leicestershire, UK; 75 Department of Infection, Immunity and Inflammation, University of Leicester, Leicester, UK; 76 Department of Clinical Epidemiology and Biostatistics, McMaster University, HSC Room 2C16, 1280 Main Street West Hamilton, Hamilton, Canada; 77 Universitätsmedizin der Johannes Gutenberg-Universität Mainz, Mainz, Germany; 78 National Heart and Lung Institute, Imperial College, London, London, UK; 79 Wellcome Centre for Global Health, Imperial College, London, London, UK; 80 MRC-PHE Centre for Environment and Health, Imperial College, London, London, UK; 81 Imperial College and Royal Brompton Hospital, London, UK; 82 Centro Medico Docente La Trinidad, Caracas, Venezuela; 83 Department of Respiratory Diseases, Montpellier University Hospital, Montpellier, France; 84 National Heart and Lung Institute, Imperial College London, Royal Brompton Hospital NHS, London, UK; 85 Institute of Ageing and Chronic Disease, University of Liverpool and University Hospital Aintree, Liverpool, UK; 86 Department of Pediatrics, Medical School, Federal University of Minas Gerais, Belo Horizonte, Brazil; 87 Région Languedoc Roussillon, Montpellier, France; 88 Department of Paediatrics, Oslo University Hospital and University of Oslo, Oslo, Norway; 89 Allergy and Asthma Associates of Southern California, Mission Viejo, CA USA; 90 Regional Ministry of Equality, Health and Social Policies of Andalusia, Seville, Spain; 91 Division of Allergy/Immunology, University of South Florida, Tampa, FL USA; 92 Allergy and Immunology Laboratory, Metropolitan University, Simon Bolivar University, Barranquilla, Colombia; 93 Asma e Immunologia, SLaai, Sociedad Latinoamericana de Allergia, Barranquilla, Colombia; 94 Department of Social Medicine, Faculty of Medicine, University of Crete, PO Box 2208, Heraklion, 71003 Crete Greece; 95 National Cooperative Group of Paediatric Research on Asthma, Asthma Clinic and Education Center of the Capital Institute of Pediatrics, Peking and Center for Asthma Research and Education, Beijing, China; 96 Chachava Clinic, David Tvildiani Medical University-AIETI Medical School, Grigol Robakidze University, Tbilisi, Georgia; 97 Pulmonolory Research Institute FMBA, Moscow, Russia; 98 National Heart and Lung Institute, Imperial College, London, London, UK; 99 Medicine Department, IRCCS-Azienda Ospedaliera Universitaria San Martino, Genoa, Italy; 100 Latvian Allergy Association, Riga, Latvia; 101 Department of Medicine, Nova Southeastern University, Davie, FL USA; 102 Department of Paediatrics, Imperial College London, London, UK; 103 The Centre for Allergy Research, The Institute of Environmental Medicine, Karolinska Institutet, Stockholm, Sweden; 104 Department of Dermatology and Allergy, Technische Universität München, Munich, Germany; 105 ZAUM-Center for Allergy and Environment, Helmholtz Center Munich, Technische Universität München, Munich, Germany; 106 Allergy Division, Chest Disease Department, University Hospital of Strasbourg, Strasbourg, France; 107 EUREGHA, European Regional and Local Health Association, Brussels, Belgium; 108 University of Edinburgh, Edinburgh, UK; 109 Allergology and Immunology Discipline, “Iuliu Hatieganu” University of Medicine and Pharmacy, Cluj-Napoca, Romania; 110 Kronikgune, Bilbao, Basque Region Spain; 111 Department of Medicine, Division of Clinical Immunology and Allergy, McMaster University, Hamilton, ON Canada; 112 Laboratoire de Pharmacologie Respiratoire UPRES EA220, Hôpital Foch, Suresnes Université Versailles Saint-Quentin, Suresnes, France; 113 Respiratory Diseases Department, Rangueil-Larrey Hospital, Toulouse, France; 114 Service de physiologie respiratoire, Hôpital Cochin, Université Paris-Descartes, Assistance publique-Hôpitaux de Paris, Paris, France; 115 NIHR Southampton Respiratory Biomedical Research Unit, Faculty of Medicine, University Southampton, Southampton, UK; 116 Medical Faculty Skopje, University Clinic of Pulmonology and Allergy, Skopje, Republic of Macedonia; 117 Service de Pneumo-Allergologie, Centre Hospitalo-Universitaire de Béni-Messous, Algers, Algeria; 118 Ecole des Mines, Alès, France; 119 Medical Faculty, Vilnius University, Vilnius, Lithuania; 120 Allergy and Clinical Immunology Section, National Heart and Lung Institute, Imperial College London, London, UK; 121 Section of Allergy and Immunology, Saint Louis University School of Medicine, Saint Louis, MO USA; 122 Pediatric Allergy and Immunology Unit, Ain Shams University, Cairo, Egypt; 123 Clinic of Children’s Diseases, Faculty of Medicine, Vilnius University, Vilnius, Lithuania; 124 Modena University, Modena, Italy; 125 Education for Health, Warwick, UK; 126 Division of Allergy, Department of Pediatric Medicine, The Bambino Gesù Children’s Research Hospital Holy See, Rome, Italy; 127 Global Allergy and Asthma Platform (GAAPP), Altgasse 8-10, 1130 Vienna, Austria; 128 Center for Health Technology and Services Research - CINTESIS, Faculdade de Medicina, Universidade do Porto, Porto, Portugal; 129 Allergy Unit, CUF Porto Instituto & Hospital, Porto, Portugal; 130 Department of Otorhinolaryngology, Academic Medical Centre, Amsterdam, The Netherlands; 131 Department of Epidemiology, Regional Health Service Lazio Region, Rome, Italy; 132 Repatriation General Hospital, Adelaide, SOUTH AUSTRALIA Australia; 133 Athens Chest Hospital, Athens, Greece; 134 National Center for Disease Control and Public Health of Georgia, Tbilisi, Georgia; 135 Polibienestar Research Institute, University of Valencia, Valencia, Spain; 136 Department of Pulmonary Diseases, Cerrahpasa Faculty of Medicine, Istanbul University, Istanbul, Turkey; 137 Allergy and Immunology Division, Clinica Ricardo Palma, Lima, Peru; 138 Universidad Autónoma de Nuevo León, San Nicolás De La Garza, Mexico; 139 Center of Allergy and Immunology, Georgian Association of Allergology and Clinical Immunology, Tbilisi, Georgia; 140 Latvian Association of Allergists, Center of Tuberculosis and Lung Diseases, Riga, Latvia; 141 Faculty of the Department of Neurology, University of Washington School of Medicine, St. Louis, MO USA; 142 University Hospital Olomouc – National eHealth Centre, Olomouc, Czech Republic; 143 Immunology and Allergy Division, Clinical Hospital, University of Chile, Santiago, Chile; 144 Centre for Infection and Immunity, School of Medicine, Dentistry and Biomedical Sciences, Queen’s University Belfast, Belfast, UK; 145 Department of Respiratory Diseases, Odense University Hospital, Odense, Denmark; 146 NHS Scotland, Edinburgh, Scotland, UK; 147 Institute of Epidemiology I, German Research Centre for Environmental Health, Helmholtz Zentrum München, Neuherberg, Germany; 148 Agence Régionale de Santé, 34067 Montpellier Cedex 2, France; 149 Vienna Challenge Chamber, Vienna, Austria; 150 Department of Paediatrics and Child Health, University College Cork, Cork, Ireland; 151 University of Southampton Faculty of Medicine, University Hospital Southampton, Southampton, UK; 152 Service de Pneumologie, Hôpital Bicêtre, Inserm UMR_S999, Université Paris-Sud, Le Kremlin Bicêtre, France; 153 School of Psychology, Plymouth University, Plymouth, UK; 154 Federico II University Hospital/Campania RS, Naples, Italy; 155 Servicio de Alergia e Immunologia, Clinica Santa Isabel, Buenos Aires, Argentina; 156 Universidade Federal de Sao Paulo, São Paulo, Brazil; 157 Libra Foundation, Buenos Aires, Argentina; 158 The George Institute for Global Health, The University of Sydney, Camperdown, Australia; 159 Airway Disease Infection Section, National Heart and Lung Institute, Imperial College, London, London, UK; 160 MRC and Asthma UK Centre in Allergic Mechanisms of Asthma, London, UK; 161 Medical Commission, Montpellier University Hospital, Montpellier, France; 162 Children’s Clinic of Tartu University Hospital, Tartu, Estonia; 163 Hallym University College of Medicine, Hallym University Sacred Heart Hospital, Gyeonggi-Do, South Korea; 164 Allergology Department, Centre de l’Asthme et des Allergies, Hôpital d’Enfants Armand-Trousseau (APHP), Paris, France; 165 Sorbonne Universités, UPMC Univ Paris 06, UMR_S 1136, Institut Pierre Louis d’Epidémiologie et de Santé Publique, Equipe EPAR, 75013 Paris, France; 166 Ukrainian Medical Stomatological Academy, Poltava, Ukraine; 167 Federal Medicobiological Agency, Laboratory of Molecular Immunology, Institute of Immunology, National Research Center, Moscow, Russian Federation; 168 Pediatric Allergy and Asthma Unit, Hacettepe University School of Medicine, Ankara, Turkey; 169 Immunology and Allergy Division, Department of Chest Diseases, School of Medicine, Hacettepe University, Ankara, Turkey; 170 Institute of Social Medicine, Epidemiology and Health Economics, Charité - Universitätsmedizin Berlin, Berlin, Germany; 171 Institute for Clinical Epidemiology and Biometry, University of Würzburg, Würzburg, Germany; 172 Department of Medicine, McMaster University, Health Sciences Centre 3V47, 1280 Main Street West, Hamilton, Canada; 173 Center for Rhinology and Allergology, Wiesbaden, Germany; 174 Société de Pneumologie de Langue Française, Espace francophone de Pneumologie, Paris, France; 175 Department of Respiratory Medicine, Faculty of Medicine and Dentistry, University Hospital Olomouc, Olomouc, Czech Republic; 176 GRIACResearch Institute, Department of Pediatric Pulmonology and Pediatric Allergology, Beatrix Children’s Hospital, University Medical Center Groningen, University of Groningen, Groningen, The Netherlands; 177 Department of Immunology, Rheumatology and Allergy, Medical University of Lodz, and HARC, Lodz, Poland; 178 Sachs’ Children’s Hospital, Stockholm, Sweden; 179 Institute of Environmental Medicine, Karolinska Institutet, Stockholm, Sweden; 180 Division of Internal Medicine, Asthma and Allergy, Barlicki University Hospital, Medical University of Lodz, Lodz, Poland; 181 Clinic of Infectious, Chest Diseases, Dermatology and Allergology, Vilnius University, Vilnius, Lithuania; 182 VIB Inflammation Research Center, Ghent University, Ghent, Belgium; 183 Department for Pediatric Pneumology and Immunology, Charité Medical University, Berlin, Germany; 184 Clínica de Alergia, Asma y Pediatría, Hospital Médica Sur, Ciudad De México, Mexico; 185 University of Medicine and Pharmacy, Hochiminh City, Vietnam; 186 Divisions of Allergy and Immunology, Departments of Internal Medicine and Pediatrics, University of Tennessee College of Medicine, Germantown, TN USA; 187 Scottish Centre for Respiratory Research, Cardiovascular and Diabetes Medicine, Medical Research Institute, Ninewells Hospital, University of Dundee, Dundee, UK; 188 State Key Laboratory of Respiratory Diseases, Guangzhou Institute of Respiratory Disease, The First Affiliated Hospital of Guangzhou Medical University, Guangzhou, 510120 China; 189 Department of Paediatrics, Oslo University Hospital, Oslo, Norway; 190 Faculty of Medicine, Institute of Clinical Medicine, University of Oslo, Oslo, Norway; 191 Department of Pulmonary Medicine, CHU Sart-Tilman, Liege, Belgium; 192 Queen’s Medical Research Institute, University of Edinburgh, Edinburgh, UK; 193 Service de Pneumo-allergologie, Hôpital Saint-Joseph, Paris, France; 194 Service de Pneumologie, UMR INSERM, UMR1087and CNR 6291, l’institut du thorax, University of Nantes, Nantes, France; 195 Department of Pulmonary Medicine, Rashid Hospital, Dubai, UAE; 196 Scottish Government Health Department, eHealth and Pharmaceuticals, Edinburgh, UK; 197 Department of Respiratory Medicine, University of Bratislava, Bratislava, Slovakia; 198 Department of Medicine (RCSI), Bon Secours Hospital, Glasnevin, Dublin, Ireland; 199 Cardiovascular and Thoracic Department, AOU Città della Salute e della Scienza di Torino, Turin, Italy; 200 Division of Clinical Immunology and Allergy, Laboratory of Behavioral Immunology Research, The University of Mississippi Medical Center, Jackson, MS USA; 201 Respiratory Medicine Research, Shahid Beheshti University of Medical Sciences, Tehran, Iran; 202 VingCard Elsafe, Moss, Norway; 203 Allergie-Centrum-Charité at the Department of Dermatology and Allergy, Charité - Universitätsmedizin Berlin, Berlin, Germany; 204 Department of Paediatrics, Maputo Central Hospital, Maputo, Mozambique; 205 Institute of Environmental Medicine, Karolinska Institutet, Stockholm, Sweden; 206 PNDR/Portuguese National Programme for Respiratory Diseases, Lisbon, Portugal; 207 Allergy and Asthma Medical Group and Research Center, San Diego, CA USA; 208 Royal Brompton Hospital, London, UK; 209 Hautklinik - Klinik für Dermatologie & Allergologie, Universitätsklinikum der RWTH Aachen, Aachen, Germany; 210 Croatian Pulmonary Society, Zagreb, Croatia; 211 National Institute of Pneumology M. Nasta, Bucharest, Romania; 212 Faculty of Medicine, University of Belgrade, Belgrade, Serbia; 213 Serbian Association for Asthma and COPD, Belgrade, Serbia; 214 National Center for Research in Chronic Respiratory Diseases, Tishreen University School of Medicine, Latakia, Syria; 215 Département de Pharmacologie, CHU de Bordeaux, Universite Bordeaux, INSERM U657, Bordeaux Cedex, France; 216 Department of Public Health and Biostatistics, EA 4064, Paris Descartes University, Paris, France; 217 Paris Municipal Department of Social Action, Childhood, and Health, Paris, France; 218 Aura Andalucia, Andalucía, Spain; 219 Allergy and Clinical Immunology Department, Hospital CUF-Descobertas, Lisbon, Portugal; 220 National Clinical Director for Respiratory Services, NHS England, Leeds, England, UK; 221 Institute of Medical Statistics, Informatics and Epidemiology, Medical Faculty, University of Cologne, Cologne, Germany; 222 Unitat de Rinologia i Clínica de l’Olfacte, Servei d’ORL, Hospital Clínic, Clinical and Experimental Respiratory Immunoallergy, IDIBAPS, Barcelona, Spain; 223 Mustapha Hospital, Algers, Algeria; 224 Scientific Centre of Children’s Health Under the Russian Academy of Medical Sciences, Moscow, Russia; 225 Section of Otolaryngology-Head and Neck Surgery, The University of Chicago Medical Center and The Pritzker School of Medicine, The University of Chicago, Chicago, IL USA; 226 Hospital de Niños Orlando Alassia, Santa Fe, Argentina; 227 Hospital of the Hospitaller Brothers in Buda, Budapest, Hungary; 228 Pediatric Pneumology and Immunology, Charité Universitätsmedizin Berlin, Berlin, Germany; 229 EA4556 Epsylon, Université Montpellier 1, Montpellier, France; 230 ENT Department, University Hospital of Kinshasa, Kinshasa, Congo; 231 Department of Allergy, Immunology and Respiratory Medicine, Alfred Hospital and Central Clinical School, Monash University, Melbourne, VIC Australia; 232 Department of Immunology, Monash University, Melbourne, VIC Australia; 233 National Hospital Organization, Tokyo National Hospital, Tokyo, Japan; 234 Department of Otorhinolaryngology, Chiba University Hospital, Chiba, Japan; 235 Department of Otolaryngology, Nippon Medical School, Tokyo, Japan; 236 Centre Hospitalier Universitaire Pédiatrique Charles de Gaulle, Ouagadougou, Burkina Faso; 237 Cardio-Thoracic and Vascular Department, University Hospital of Pisa, Pisa, Italy; 238 Department of Comparative Medicine, Messerli, Research Institute of the University of Veterinary Medicine and Medical University, Vienna, Austria; 239 Department of Immunology and Allergology, Faculty of Medicine and Faculty Hospital in Pilsen, Charles University in Prague, Pilsen, Czech Republic; 240 Center for Pediatrics and Child Health, Institute of Human Development, Royal Manchester Children’s Hospital, University of Manchester, Manchester, M13 9WL UK; 241 Allergy Department, 2nd Pediatric Clinic, Athens General Children’s Hospital “P&A Kyriakou”, University of Athens, Athens, 11527 Greece; 242 Respiratory Medicine, Department of Medical Sciences, University of Ferrara, Ferrara, Italy; 243 Department of Allergy and Clinical Immunology, Ajou University School of Medicine, Suwon, South Korea; 244 Nuffield Department of Medicine, University of Oxford, Oxford, UK; 245 Department of Pediatrics, Nippon Medical School, Tokyo, Japan; 246 Department of Health, Social Services and Public Safety, Belfast, Northern Ireland, UK; 247 Center for Rhinology and Allergology, Wiesbaden, Germany; 248 Department of Otorhinolaryngology, Head and Neck Surgery, Universitätsmedizin Mannheim, Medical Faculty Mannheim, Heidelberg University, Mannheim, Germany; 249 Conseil Général de l’Economie. Ministère de l’Economie, de l’Industrie et du Numérique, Paris, France; 250 Département de pédiatrie, CHU de Grenoble, BP 217, 38043 Grenoble Cedex 9, France; 251 Children’s Hospital Srebrnjak, Zagreb, School of Medicine, University J.J. Strossmayer, Osijek, Croatia; 252 Im GerontoLab Europe - Europäische Vereinigung für Vitalität und Aktives Altern (eVAA) e.V., Leipzig, Germany; 253 Karl Landsteiner Institute for Clinical and Experimental Pneumology, Hietzing Hospital, Wolkersbergenstraße 1, 1130 Vienna, Austria; 254 Clinic of Allergy and Asthma, Medical University Sofia, 1Sv. Georgi Sofiyski St., 1431 Sofia, Bulgaria; 255 Allergy Diagnostic and Clinical Research Unit, University of Cape Town Lung Institute, Cape Town, South Africa; 256 Department of Pulmonary Medicine and Tuberculosis, GRIAC Research Institute, University Medical Center Groningen, University of Groningen, Groningen, The Netherlands; 257 Academic Centre of Primary Care, University of Aberdeen, Aberdeen, UK; 258 Research in Real-Life, Cambridge, UK; 259 LungenClinic Grosshansdorf, Airway Research Center North, Member of the German Center for Lung Research (DZL), Grosshansdorf, Germany; 260 Department of Medicine, Christian Albrechts University, Airway Research Center North, Member of the German Center for Lung Research (DZL), Kiel, Germany; 261 Conseil Départemental de l’Ordre des Pharmaciens, Maison des Professions Libérales, 34000 Montpellier, France; 262 SLAAI, Barranquilla, Colombia; 263 Division of Pulmonary, Critical Care, Sleep and Allergy, University of Nebraska Medical Center, Omaha, NE USA; 264 Salford, Royal NHS Foundation Trust and NHS England North, London, UK; 265 Hospital Universitario de Getafe-Servicio Madrileño de Salud, Madrid, Spain; 266 Association Asthme et Allergie, Paris, France; 267 Primary Care Respiratory Research Unit, Institutode Investigación Sanitaria de Palma IdisPa, Palma De Mallorca, Spain; 268 Allergy Unit, Complesso integrato Columbus, Rome, Italy; 269 Serviço de Imunoalergologia, Hospital da Luz, Lisbon, Portugal; 270 Hospital de Clinicas, University of Parana, Curitiba, PR Brazil; 271 Department of Allergy, Asthma, and Immunology, Children’s Mercy Hospitals and Clinics and Pediatrics, Medicine University of Misouri-Kansas City School of Medicine, Kansas City, MO USA; 272 Division of Allergy Asthma and Clinical Immunology, Emek Medical Center, Afula, Israel; 273 Woodbrook Medical Centre, Loughborough, UK; 274 Honorary Clinical Research Fellow, Allergy and Respiratory Research Group, The University of Edinburgh, Edinburgh, UK; 275 Allergy and Clinical Immunology Department, Centro Médico-Docente la, Trinidad and Clínica El Avila, 6a transversal Urb, Altamira, piso 8, consultorio 803, Caracas, 1060 Venezuela; 276 The Royal National TNE Hospital, University College London, London, UK; 277 Otolaryngology and Head and Neck Surgery, CHU Rangueil-Larrey, Toulouse, France; 278 Allergy Unit, Department of Dermatology, University Hospital of Zurich, Zurich, Switzerland; 279 Helmholtz Zentrum München/Institute of Epidemiology I, Neuherberg, Germany; 280 Allergy and Respiratory Research Group, Centre for Population Health Sciences, Medical School, The University of Edinburgh, Edinburgh, UK; 281 Child Health, Queen’s University Belfast and Royal Belfast Hospital for Sick Children, Belfast, UK; 282 Department of Thoracic Medicine, University Hospital of Heraklion, Crete, Greece; 283 University Hospital of Mont-Godinne, Catholic University of Louvain, Yvoir, Belgium; 284 Sorbonne Universités, UPMC Univ Paris 06, UMR_S 1158 Neurophysiologie Respiratoire Expérimentale et Clinique, Paris, France; 285 INSERM, UMR_S 1158 Neurophysiologie Respiratoire Expérimentale et Clinique, Paris, France; 286 Department R3S, AP-HP, Groupe, Paris, France; 287 Department of Pediatrics and Child Health, Department of Immunology, Faculty of Medicine, University of Manitoba, Winnipeg, MB Canada; 288 Sociedad Paraguaya de Alergia Asma e Inmunología, Asunción, Paraguay; 289 Julius Center of Health Sciences and Primary Care, University Medical Center Utrecht, University of Utrecht, Utrecht, The Netherlands; 290 Division of Allergy, Clinical Immunology and Rheumatology, Department of Pediatrics, Federal University of São Paulo, São Paulo, Brazil; 291 Kyrgyzstan National Centre of Cardiology and Internal Medicine, Euro-Asian Respiratory Society, Bishkek, Kyrgyzstan; 292 Pulmonary Division, Heart Institute (InCor), Hospital da Clinicas da Faculdade de Medicina da Universidade de Sao Paulo, São Paulo, Brazil; 293 Academic Medical Centre, University of Amsterdam, Amsterdam, The Netherlands; 294 Department of Epidemiology, CAPHRI School of Public Health and Primary Care, Maastricht University, Maastricht, The Netherlands; 295 Sidkkids hospitala and Institute of Health Policy, Management and Evaluation, Toronto, ON Canada; 296 Centre of Pneumology, Faculty of Medicine, University of Coimbra, Coimbra, Portugal; 297 Division of Allergy and Clinical Immunology, University of Salerno, Salerno, Italy; 298 Division of Immunopathology, Department of Pathophysiology and Allergy Research, Center for Pathophysiology, Infectiology and Immunology, Medical University of Vienna, Vienna, Austria; 299 Pneumology and Allergy Department, Hospital Clínic, Clinical and Experimental Respiratory Immunoallergy, IDIBAPS, Barcelona, Spain; 300 Department of Lung Diseases and Clinical Allergology, University of Turku, Turku, Finland; 301 Unité de Pharmacoépidémiologie, CHU-Lyon - UR 5558 CNRS, Université Claude Bernard, Lyon, Villeurbanne, France; 302 Clinical Immunology and Allergy Unit, Department of Medicine Solna, Karolinska Institutet and University Hospital, Stockholm, Sweden; 303 Department of Chest Medicine, Centre Hospitalier Universitaire Dinant-Godinne, Université Catholique de Louvain, Yvoir, Belgium; 304 FILHA, Finnish Lung Association, Helsinki, Finland; 305 Gérontopôle, CHU Toulouse, Toulouse, France; 306 Centre for Respiratory Medicine and Allergy, Institute of Inflammation and Repair, Manchester Academic Health Science Centre, The University of Manchester, Manchester, UK; 307 University Hospital of South Manchester, Manchester NHS Foundation Trust, Manchester, UK; 308 Pulmonary Unit, Department of Cardiology, Thoracic and Vascular Medicine, Arcispedale S.Maria Nuova/IRCCS, Research Hospital, Reggio Emilia, Italy; 309 Regional Agency for Health and Social Care, Reggio Emilia, Italy; 310 Division of Allergy and Immunology, Department of Pediatrics, Siriraj Hospital, Mahidol University Faculty of Medicine, Bangkok, 10700 Thailand; 311 Pulmonary Environmental Epidemiology Unit, CNR Institute of Clinical Physiology, Pisa, Via Trieste 41, 56126 Pisa, Italy; 312 CNR Institute of Biomedicine and Molecular Immunology “A. Monroy”, Via U. La Malfa 153, 90146 Palermo, Italy; 313 Department of Medicine, Pulmonary and Critical Care Medicine, University Medical Center Giessen and Marburg, Philipps-University Marburg, Marburg, Germany; 314 Sotiria Hospital, Athens, Greece; 315 Department of Otorhinolaryngology, HNO-Klinik, Universitätsklinikum Düsseldorf, Düsseldorf, Germany; 316 Hôpital Albert Calmette, CHRU, Lille, France; 317 Asthma UK, Mansell Street, London, UK; 318 Department of Otolaryngology, Yong Loo Lin School of Medicine, National University of Singapore, Singapore, Singapore; 319 Eshelman School of Pharmacy, University of North Carolina, Chapel Hill, NC USA; 320 Bradford Institute for Health Research, Bradford Royal Infirmary, Bradford, UK; 321 Department of Research, Olmsted Medical Center, Rochester, MN USA; 322 Cyprus International Institute for Environmental and Public Health in Association with Harvard School of Public Health, Cyprus University of Technology, Limassol, Cyprus; 323 Department of Pediatrics, Hospital “Archbishop Makarios III”, Nicosia, Cyprus; 324 The Allergy and Asthma Institute, Lahore, Pakistan; 325 Social Sciences, University of Southampton, Southampton, UK; 326 Department of Paediatrics and Child Health, Red Cross Children’s Hospital, and MRC Unit on Child and Adolescent Health, University of Cape Town, Cape Town, South Africa; 327 Universidad Católica de Córdoba, Córdoba, Argentina; 328 Department of Otolaryngology, Head and Neck Surgery, Beijing Tongren Hospital, Capital Medical University, Beijing, 100730 China; 329 University Clinic of Respiratory and Allergic Diseases, Golnik, Slovenia; 330 Department of Physiology, CHRU and Vice President for Research, University Montpellier, Montpellier, France; 331 EPAR UMR-S UPMC, Paris VI, Paris, France

## Erratum to: Clin Transl Allergy (2016) 6:29 DOI 10.1186/s13601-016-0116-9

In this version of this article that was originally published [1], the name M. R. Khaitov was misspelled as M. R. Kaitov.
