# Supplementary material for: Scaling up strategies of the chronic respiratory disease programme of the European Innovation Partnership on Active and Healthy Ageing (Action Plan B3: Area 5)
Source: Clin Transl Allergy. 2016 Jul 29;6:29. doi: 10.1186/s13601-016-0116-9 (PMC4966705; doi:10.1186/s13601-016-0116-9)
Supplement: Supplementary file 1 — 10.1186/s13601-016-0116-9 IPCRG scaling up activities. [file 13601_2016_116_MOESM1_ESM.docx]

**Supplement 1: IPCRG scaling up activities**

| **Strategy papers** | **Description of resource** | **Link to European resource/contact** | **Link to outside Europe resource or contact** |  |  |
| --- | --- | --- | --- | --- | --- |
| Primary care strengthening | Referenced position paper on primary care and respiratory disease | <http://www.theipcrg.org/display/OurNetwork/POSITION+PAPER+1+Primary+care+and+chronic+lung+disease> | <http://www.theipcrg.org/display/OurNetwork/POSITION+PAPER+1+Primary+care+and+chronic+lung+disease> |  |  |
| Education for primary care clinicians about respiratory care: what works | McDonnell J, Williams S, Chavannes NH, de Sousa JC, Fardy HJ, Fletcher M et al. Effecting change in primary care management of respiratory conditions: a global scoping exercise and literature review of educational interventions to inform the IPCRG's E-Quality initiative. Prim Care Respir J. 2012 Dec;21(4):431-436. | Available from: 10.4104/pcrj.2012.00071 and IPCRG website http://www.theipcrg.org/display/TeachColleagues/Teaching+colleagues | Available from: 10.4104/pcrj.2012.00071 and IPCRG website http://www.theipcrg.org/display/TeachColleagues/Teaching+colleagues |  |  |
| Education for respiratory clinicians: how to build capacity | McDonnell J, Correia de Sousa J, Baxter N, Pinnock H, Roman-Rodriquez M, van der Molen T, Williams S. Building capacity to improve respiratory care: the education strategy of the International Primary Care Respiratory Group 2014–2020 | http://www.nature.com/articles/npjpcrm201472 and more information from http://www.theipcrg.org/display/TeachColleagues/Teaching+colleagues | http://www.nature.com/articles/npjpcrm201472 and more information from http://www.theipcrg.org/display/TeachColleagues/Teaching+colleagues |  |  |
| Research questions to meet primary care practitioner needs | Pinnock H, Ostrem A, Rodriguez MR, Ryan D, Stallberg B, Thomas M, Tsiligianni I, Williams S, Yusuf O. The International Primary Care Respiratory Group (IPCRG) Research Needs Statement 2010. | http://www.nature.com/articles/pcrj201021#abstract more information from http://www.theipcrg.org/display/DoResearch/Prioritised+primary+care+respiratory+research+questions+from+our+reseach+needs+statement | http://www.nature.com/articles/pcrj201021#abstract more information from http://www.theipcrg.org/display/DoResearch/Prioritised+primary+care+respiratory+research+questions+from+our+reseach+needs+statement |  |  |
| Research questions to meet primary care practitioner needs (prioritised) | Pinnock H et al. Prioritising the respiratory research needs of primary care: the International Primary Care Respiratory Group (IPCRG) e-Delphi exercise. Prim Care Respir J 2012;21(1):19-27. | http://www.nature.com/articles/pcrj20126 and more information from http://www.theipcrg.org/display/DoResearch/Prioritised+primary+care+respiratory+research+questions+from+our+reseach+needs+statement | http://www.nature.com/articles/pcrj20126 and more information from http://www.theipcrg.org/display/DoResearch/Prioritised+primary+care+respiratory+research+questions+from+our+reseach+needs+statement |  |  |
|  |  |  |  |  |  |
| **Chronic respiratory disease** | **Description of resource** | **Link to European resource/contact** | **Link to outside Europe resource or contact** |  |  |
| Health protection: immunisation | Review of the literature on respiratory disease and immunisation. | Will be submitted for publication by December 2015 and a short online version produced for IPCRG website. | Will be submitted for publication by December 2015 and a short online version produced for IPCRG website. |  |  |
| Diagnosis and management of CHRONIC RESPIRATORY DISEASE in primary care | Slidesets used for WONCA (global family doctor association) meetings from 2011 to 2014 to inform grass-roots GPs. IPCRG is the respiratory special interest group of WONCA Europe | http://www.theipcrg.org/display/wonca/WONCA+2011+Presentations http://www.theipcrg.org/display/wonca/WONCA+2012+Presentations http://www.theipcrg.org/display/wonca/WONCA+2013+Presentations http://www.theipcrg.org/display/wonca/WONCA+2014+presentations |  |  |  |
| Asthma, COPD, tobacco dependence, allergic rhinitis, sleep apnoea | Map of national guidelines 2013/14; summary; classified by country and by disease | http://ipcrg.new.quintor.nl/display/ResMapping/Asthma http://ipcrg.new.quintor.nl/pages/viewpage.action?pageId=6455884 http://www.theipcrg.org/display/ResMapping/Support+for+stopping+smoking http://ipcrg.new.quintor.nl/display/ResMapping/Allergic+rhinitis http://www.theipcrg.org/pages/viewpage.action?pageId=6455895 | http://ipcrg.new.quintor.nl/display/ResMapping/Asthma http://ipcrg.new.quintor.nl/pages/viewpage.action?pageId=6455884 http://www.theipcrg.org/display/ResMapping/Support+for+stopping+smoking http://ipcrg.new.quintor.nl/display/ResMapping/Allergic+rhinitis http://www.theipcrg.org/pages/viewpage.action?pageId=6455895 |  |  |
| Asthma | Teach the teacher programme, difficult to manage asthma including a desktop helper translated into multiple European languages | http://ipcrg.new.quintor.nl/display/TreatP/Home+-+Difficult+to+manage+asthma http://www.theipcrg.org/display/TreatP/Desktop+helper+difficult+to+manage+asthma http://www.theipcrg.org/display/TreatP/Difficult+to+manage+asthma+-+Position+Paper |  |  |  |
| Allergic rhinitis (AR) | Links to primary care resources on AR | <http://www.theipcrg.org/display/TreatP/Treating+Allergic+Rhinitis> |  |  |  |
| Tobacco dependence | Review of tobacco dependence guidelines for primary care and analysis of how evidence-based they are and how primary care was engaged | Sheals, K., Allistone, G. & McEwen A. (2014) The nature and extent of national tobacco treatment guidelines for primary care: Report for the International Primary Care Respiratory Group. London, National Centre for Smoking Cessation and Training. Final report (5 December 2014). http://www.theipcrg.org/display/RESSMO/Tobacco+treatment%3A+list+of+national+guidelines+and+scope+of+guidelines+for+primary+care | Sheals, K., Allistone, G. & McEwen A. (2014) The nature and extent of national tobacco treatment guidelines for primary care: Report for the International Primary Care Respiratory Group. London, National Centre for Smoking Cessation and Training. Final report (5 December 2014). http://www.theipcrg.org/display/RESSMO/Tobacco+treatment%3A+list+of+national+guidelines+and+scope+of+guidelines+for+primary+care |  |  |
| Tobacco dependence | Tackling the smoking epidemic set of resources including desktop helpers (due for review November 2015), Powerpoint slides and online resource | http://www.theipcrg.org/display/TreatP/Tobacco+Dependence http://www.theipcrg.org/display/TreatP/IPCRG+Opinion+3%3A+Helping+patients+quit+smoking | http://www.theipcrg.org/display/TreatP/Tobacco+Dependence http://www.theipcrg.org/display/TreatP/IPCRG+Opinion+3%3A+Helping+patients+quit+smoking |  |  |
| Chronic respiratory disease in low income countries (the FRESH AIR movement) | Progress reports and papers from the IPCRG movement to describe the prevalence of smoke from smoking cigarettes, pipes and roll-ups and from indoor air pollution generated by cooking and heating biomass fuel and kerosene lighting. Note that a new Horizon 2020 FRESH AIR programme is due to launch in October 2015. |  | Protocols for prevalence study and intervention study available on request to IPCRG http://www.theipcrg.org/display/DoResearch/FRESH+AIR http://www.theipcrg.org/display/DoResearch/About+FRESH+AIR+Uganda http://www.theipcrg.org/display/DoResearch/FRESH+AIR+India http://www.theipcrg.org/display/DoResearch/FRESH+AIR+Kyrgyzstan http://www.theipcrg.org/pages/viewpage.action?pageId=8781866 |  |  |
| COPD | Desktop "how to" helpers on palliative care, earlier diagnosis, spirometry; some in multiple languages | http://www.theipcrg.org/display/TreatP/Treating+Patients+Home http://www.theipcrg.org/display/TreatP/IPCRG+Opinion+5+-+Early+diagnosis+of+COPD http://www.theipcrg.org/display/TreatP/IPCRG+Opinion+2%3A+Theophylline+Opinion+Sheet http://www.theipcrg.org/display/TreatP/IPCRG+Opinion+1%3A+Opinion+Sheet+on+Spirometry http://www.theipcrg.org/display/TreatP/IPCRG+Opinion+4%3A+Palliative+Care+Opinion+Sheet | http://www.theipcrg.org/display/TreatP/Treating+Patients+Home http://www.theipcrg.org/display/TreatP/IPCRG+Opinion+5+-+Early+diagnosis+of+COPD http://www.theipcrg.org/display/TreatP/IPCRG+Opinion+2%3A+Theophylline+Opinion+Sheet http://www.theipcrg.org/display/TreatP/IPCRG+Opinion+1%3A+Opinion+Sheet+on+Spirometry http://www.theipcrg.org/display/TreatP/IPCRG+Opinion+4%3A+Palliative+Care+Opinion+Sheet |  |  |
| Use of routine primary care datasets to identify best practice or problems that need an intervention | IPCRG set up UNLOCK to combine data sets from routine primary care. A number of projects have now been published | <http://www.theipcrg.org/display/DoResearch/About+UNLOCK> | Colleagues from outside Europe are involved but have not yet published. |  |  |
| Mental health and respiratory disease | A review paper on mental health and respiratory disease, looking at both people with mental health problems who are at high risk of smoking and therefore respiratory problems and also people with asthma and COPD who have anxiety and depression, and the effectieness of interventions. | Review due for submission to peer-reviewed journal December 2015 and a summary position paper online for IPCRG | Review due for submission to peer-reviewed journal December 2015 and a summary position paper online for IPCRG |  |  |
